# New AKT-dependent mechanisms of anti-COVID-19 action of high-CBD *Cannabis sativa* extracts

**DOI:** 10.1038/s41420-022-00876-y

**Published:** 2022-03-11

**Authors:** Bo Wang, Dongping Li, Anna Fiselier, Igor Kovalchuk, Olga Kovalchuk

**Affiliations:** 1 Department of Biological Sciences, University of Lethbridge, Lethbridge, AB T1K 3M4 Canada; 2 Pathway Rx Inc., Lethbridge, AB T1K 3M4 Canada; 3 University of Calgary, Cumming School of Medicine, Calgary, AB T2N 1N4 Canada; 4 Swysh Inc., Lethbridge, AB T3H 4Z2 Canada

**Keywords:** Molecular biology, Epigenetics

## Abstract

COVID-19 is caused by the SARS-CoV-2 virus, which enters target cells via interactions with ACE2 and TMPRSS2. Here, we show AKT serine/threonine kinase-dependent epigenetic control of ACE2 and TMPRSS2 expression by high-cannabidiol (CBD) cannabis extracts and their individual components. CBD alone and extracts #1, #5, #7, and #129 downregulated ACE2 and TMPRSS2 in lung fibroblast WI-38 cells through AKT-mediated inhibition. miR-200c-3p and let-7a-5p were two contributing miRNAs in CBD-mediated suppression of ACE2 and TMPRSS2. CBD and terpene PTWT2.2 profoundly inhibited ACE2 and TMPRSS2 expression, both individually and in combination. Extracts #1, #5, #7, and #169 suppressed COX2 expression and remarkably attenuated TNFα/IFNγ-triggered induction of proinflammatory factors IL-6 and IL-8 by AKT pathway. The most abundant molecules present in extracts #1 and #7 modulated the expression of COX2, IL-6, and IL-8 both individually and in combination. These results reveal that high-CBD cannabis extracts attenuated ACE2 and TMPRSS2 expression and the induction of inflammatory mediators COX2, IL-6, and IL-8 via the AKT pathway, highlighting their potential anti-COVID-19 features.

## Introduction

As of this writing SARS-CoV-2-induced coronavirus disease (COVID-19) [1, 2] affected over 295 million people, and caused over 5.4 million deaths.

Patients with severe COVID-19, who account for 20% of all cases, develop serious complications, including acute respiratory distress symptoms (ARDS), as well as acute cardiac, liver, kidney, and nervous system injuries [1, 3–6]. Multi-organ failure may be attributed to excessive production of proinflammatory cytokines—the so-called “cytokine storm” [1, 3, 7–9], and interleukin 6 (IL-6) and tumor necrosis factor α (TNF-α) are the pivotal players in cytokine storm pathogenesis and COVID-19 [9, 10].

SARS-CoV-2 infects human cells using angiotensin-converting enzyme 2 (ACE2) as a functional cell-entry receptor. Other host factors, including transmembrane serine protease 2 (TMPRSS2) and neuropillin 1 (NRP1), also have roles in the infection process [11–13].

Although several SARS-CoV-2 vaccines have been approved, no therapies have yet proven effective for COVID-19 [14]. Hence, there is an urgent need to develop an effective treatment against SARS-CoV-2.

The medicinal use of cannabis and cannabinoids has a long history, although it has garnered much more attention in recent years. To date, more than 100 cannabinoids, also referred to as phytocannabinoids, have been identified in the *Cannabis sativa* plant. The two most abundant phytocannabinoids are Δ^9^-tetrahydrocannabinol (Δ^9^-THC) and cannabidiol (CBD). *C. sativa* also produces other minor cannabinoids as well as terpenes and terpenoids, which have shown modulating effects on main phytocannabinoids, often referred to as the “entourage effect” [15]. The biological effects of phytocannabinoids may primarily be mediated by cannabinoid receptors 1 and 2 (CB1R and CB2R) [16]. Accumulating evidence indicates that cannabinoids may have anti-inflammatory and anti-cancer properties mediated by reducing the production of proinflammatory cytokines and attenuating tumor growth [17, 18]. However, the mechanism involved remains poorly understood.

Our previous studies in 3D tissue models have shown that cannabis extracts downregulate the expression of ACE2, IL-6, and TNF-α, the key factors in COVID-19 progression [19, 20]. Here, using human normal cell lines as a model system, we found that cannabis extracts #1, #5, #98, and #129 inhibit ACE2 and TMPRSS2 expression, and extracts #1, #5, #7, and #169 attenuate the expression of inflammatory mediators cyclooxygenase-2 (COX2), IL-6, and interleukin 8 (IL-8), transcriptionally and/or post-transcriptionally. Our findings highlight the anti-SARS-CoV-2 host entry and anti-cytokine release syndrome properties of selected cannabis extracts, supporting the urgent need for clinical trials.

## Results

### CBD and cannabis extracts inhibit ACE2 and TMPRSS2 expression both transcriptionally and/or post-transcriptionally

**Fig. 1:**
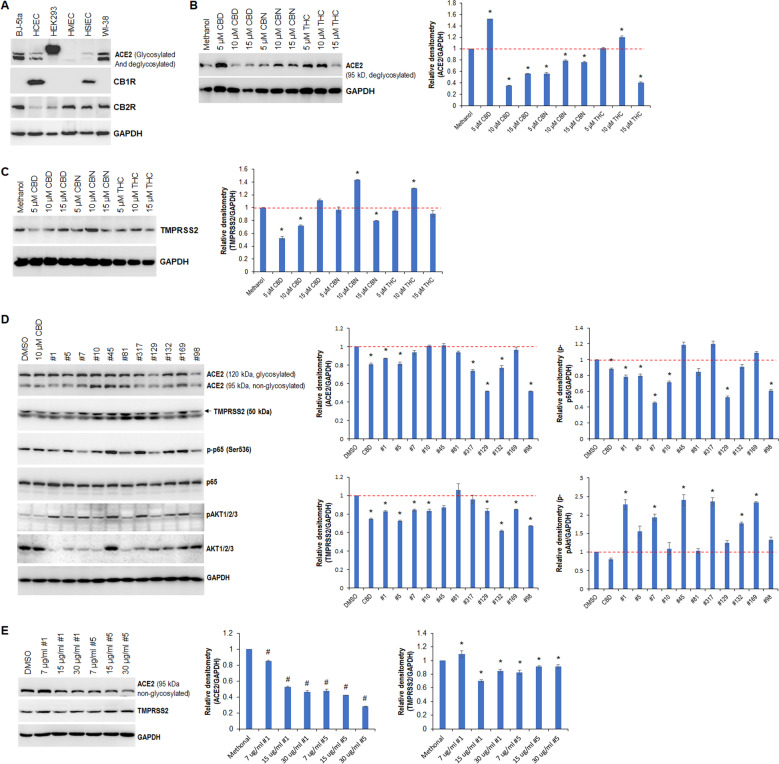
Cannabinoids and cannabis extracts modulate the expression of ACE2 and TMPRSS2 in WI-38 cells. **A** Western blot analysis of ACE2, CB1R and CB2R in the indicated cell lines, GAPDH served as a loading control. **B**, **C** Western blot analysis of ACE2 (**B**) and TMPRSS2 (**C**) in WI-38 cells treated with the indicated cannabinoids; relative densitometry was determined using ImageJ. **D** Western blot analysis of the indicated proteins in WI-38 cells treated with either CBD or the indicated cannabis extracts; relative densitometry was measured using ImageJ. **E** Western blot analysis of ACE2 and TMPRSS2 in WI-38 cells treated with the indicated extracts; relative densitometry was measured using ImageJ. * indicates *p* < 0.05; # indicates *p* < 0.001.

Our previous studies indicated that high-CBD cannabis extracts may inhibit SARS-CoV-2 infection of host cells by downregulating ACE2 and TMPRSS2 [19]. However, the mechanism remains unknown. To establish a cell model system in which the mechanism could be explored, we evaluated the expression patterns of ACE2, CB1R, and CB2R in the following normal human cell lines: foreskin fibroblasts (BJ-5ta), primary colonic epithelial cells, embryonic kidney epithelial cells, mammary epithelial cells, primary small intestinal epithelial cells, and lung fibroblasts (WI-38). Western blot analysis showed that ACE2, CB1R, and CB2R were differentially expressed in the tested cell lines (Fig. 1A). The WI-38 cell line was used as a model to screen cannabis extracts that suppress ACE2 and TMPRSS2 expression.

To determine the effect of pure cannabinoids on the expression of ACE2 and TMPRSS2, we first treated WI-38 cells with a range of CBD, cannabinol (CBN), and THC doses. Western blotting indicated that CBD, CBN, and THC differentially modulated ACE2 and TMPRSS2 expression (Fig. 1B, C). Since 10 μM CBD was able to downregulate the expression of both ACE2 and TMPRSS2, it served as a positive control. We then exposed WI-38 cells to 10 μM CBD or 15 μg/ml of various extracts. Western blot analysis showed that CBD and extracts #1, #5, # 98, #129, and #132 inhibited both ACE2 and TMPRSS2 expression (Fig. 1D). Extracts #7, #10, and #169 also inhibited TMPRSS2 expression, but they had no effect on ACE2 expression.

We also looked at the effect on signaling pathways. Western blotting showed that CBD decreased the levels of both phosphorylated p65 (p-p65, Ser536) and phosphorylated AKT1/2/3 (p-AKT1/2/3), whereas extracts #45 and #317 increased the levels of both p-p65 and p-AKT1/2/3 (Fig. 1D). Interestingly, extracts #1, #5, #7, #98, and #129 downregulated p-p65, while they upregulated p-AKT1/2/3 (Fig. 1D). Although extracts #10 and #81 decreased p-p65, they had no effect on p-AKT1/2/3. Extracts #132 and #169 upregulated p-AKT1/2/3; however, they had no effect on p-p65. To determine whether the effect on ACE2 and TMPRSS2 expression was dose-dependent, WI-38 cells were exposed to a range of doses of extracts #1 and #5. Western blot analysis indicated a dose-dependent effect on ACE2 expression in response to both extracts (Fig. 1E); however, the effect on TMPRSS2 expression was less dose-dependent.

**Fig. 2:**
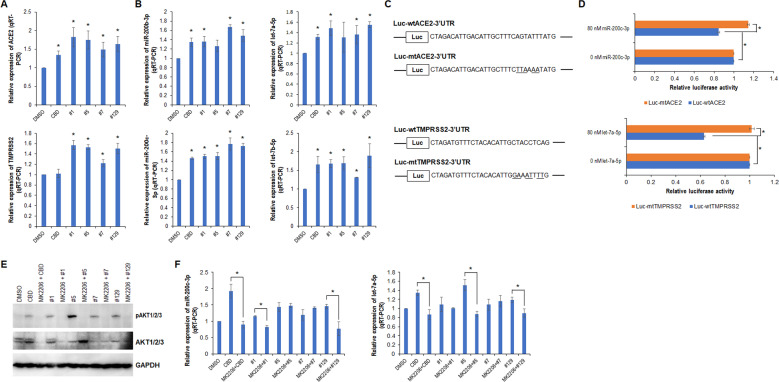
CBD and cannabis extracts enhance expression of miR-200c-3p and let-7a-5p through AKT pathway. **A**, **B** qRT-PCR analysis of miR-200b-3p, miR-200c-3p, let-7a-5p and let-7b-5p in WI-38 cells treated with either CBD or the selected extracts. **C** Diagram of luciferase reporter bearing either wild-type or mutant ACE2 3′UTR or TMPRSS2 3′UTR; the mutated deoxy nucleotides are underlined. **D** Luciferase assay was performed as described in the Methods section. **E** Western blot analysis of pAKT1/2/3 and AKT1/2/3 in WI-38 cells treated with MK2206 for 2 h prior to the addition of CBD or the selected extracts. **F** qRT-PCR analysis of miR-200c-3p or let-7a-5p in WI-38 cells treated with MK2206 for 2 h prior to the addition of CBD or the selected extracts. * indicates *p* < 0.05.

To explore the mechanism underlying the extract-mediated downregulation of ACE2 and TMPRSS2, we measured the mRNA levels of the two genes that encode ACE2 and TMPRSS2. The quantitative real-time RT-PCR (qRT-PCR) showed that extracts #1, #5, #7, and #129 enhanced transcription of *ACE2* and *TMPRSS2* (Fig. 2A). CBD alone also slightly activated the transcription of *ACE2*, while it had no effect on *TMPRSS2* transcription. Since the extract-induced transcriptional changes to *ACE2* and *TMPRSS2* genes were inversely correlated with the respective protein levels, this implicated the involvement of the post-transcriptional mechanism.

Bioinformatic analysis predicted that miR-200b/c-3p and miR-429 could target the *ACE2* 3′UTR, and that miR-4458, miR-4500, and let-7 family members could target the *TMPRSS2* 3′UTR (Figs. S1, S2). The target sequences are highly conserved among different species. To investigate the role of miRNAs in the extract-mediated downregulation of ACE2 and TMPRSS2, we determined the expression levels of selected miRNAs. The qRT-PCR showed that miR-200b-3p, miR-200c-3p, let-7a-5p, and let-7b-5p were all upregulated in WI-38 cells in response to CBD and extracts #1, #5, #7, and #129 (Fig. 2B). This suggested that the miRNAs may have roles in post-transcriptional targeting of ACE2 and TMPRSS2. To confirm our hypothesis, we constructed luciferase reporters bearing either wild-type or mutant targeting sequences (Fig. 2C). A luciferase assay showed that miR-200c-3p and let-7a-5p significantly attenuated the luciferase activity of reporters carrying wild-type *ACE2* 3′UTR and *TMPRSS2* 3′UTR, respectively; the mutant reporter did not respond to miR-200c-3p and let-7a-5p (Fig. 2D).

To determine whether AKT pathway activation plays a role in the induction of miR-200c-3p and let-7a-5p mediated by extracts, WI-38 cells were pretreated with 80 nM MK2206, a highly selective AKT1/2/3 inhibitor. Western blot analysis and qRT-PCR indicated that the induction of miR-200c-3p by CBD and extracts #1 and #129 was blocked by MK2206, while induction by extracts #5 and #7 was not. Similarly, the induction of let-7a-5p was blocked by MK2206 in response to CBD and extracts #5 and #129, but not to extracts #1 and #7 (Fig. 2E, F). In the loss-of-function studies, inhibitors of miR-200c-3p and let-7a-5p significantly restored both ACE2 and TMPRSS2 expression that was downregulated by CBD, and rescued the ACE2 expression that was downregulated by extracts #1 and #129 (Fig. S3), supporting a role of these miRNAs in CBD and extracts #1 and #129-mediated epigenetic silencing of ACE2 and/or TMPRSS2.

To confirm our findings, we repeated the experiments in another fibroblast cell line, BJ-5ta, which showed a similar ACE2, CB1R, and CB2R expression pattern to WI-38 (Fig. 1A). BJ-5ta cells were treated with either 10 μM CBD or 15 μg/ml of the indicated extracts. Western blot analysis showed that CBD and extracts #1, #5, #7, #98, and #129 downregulated ACE2 and TMPRSS2 expression (Fig. S4) and reduced p-p65 levels (Ser536, Fig. S3). These results were consistent with those observed in WI-38 cells. Interestingly, extracts #1, #5, #7, #98, and #129 decreased pAKT1/2/3 levels in BJ-5ta cells (Fig. S4), whereas they increased its levels in WI-38 cells (Fig. 1D).

**Fig. 3:**
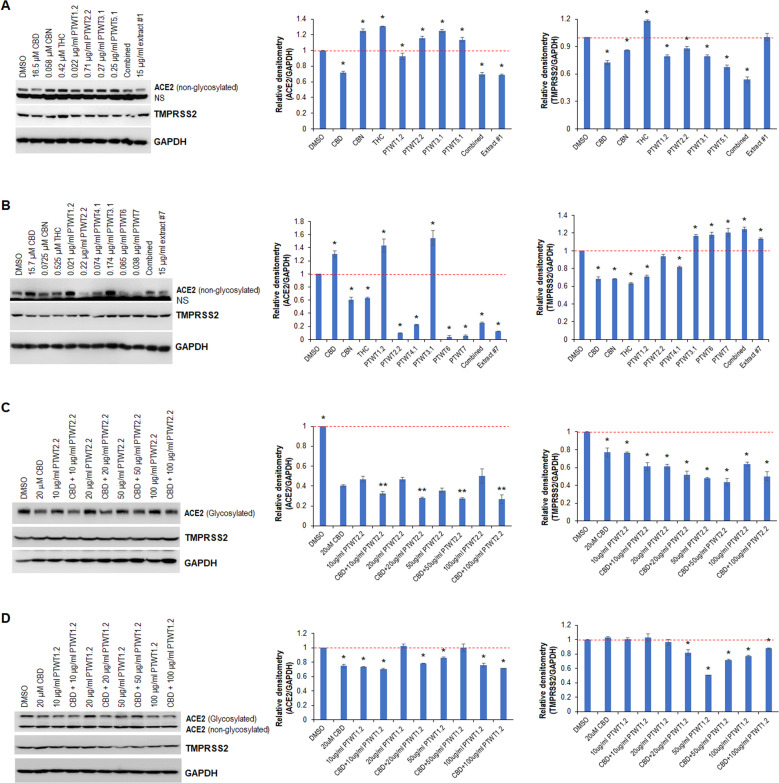
The most abundant compounds in extracts #1 and #5 modulate the expression of ACE2 and TMPRSS2 individually and collaboratively. **A** Western blot analysis of ACE2 and TMPRSS2 in WI-38 cells treated with the indicated most abundant compounds in extract #1, with the same concentration found in the extract; relative densitometry was measured using ImageJ. **B** Western blot analysis of ACE2 and TMPRSS2 in BJ-5ta cells treated with the indicated most abundant compounds in extract #7, with the same concentration found in the extract; relative densitometry was measured using ImageJ. **C** Western blot analysis of ACE2 and TMPRSS2 in WI-38 cells treated with either CBD or PTWT2.2 individually or in combination; relative densitometry was measured using ImageJ. **D** Western blot analysis of ACE2 and TMPRSS2 in WI-38 cells treated with either CBD or PTWT1.2 individually or in combination; relative densitometry was measured using ImageJ. PTWT represents terpene. * indicates *p* < 0.05; ** indicates *p* < 0.01.

Next, we examined the effects of the most abundant compounds in extracts #1 and #7 on ACE2 and TMPRSS2 expression. For this, WI-38 and BJ-5ta cells were treated with either CBD, CBN, THC, or terpenes individually or in combination, using the same concentrations found in extracts #1 and #7. Western blotting indicated that CBD, PTWT1.2, a combination of all main terpenes, and extract #1 significantly downregulated ACE2 expression, while other compounds tested upregulated ACE2 expression (Fig. 3A, *p* < 0.05). The effects of extract #1 and the ‘combined’ treatment were similarly effective and were not different from each other. All treatments, except THC and extract #1, reduced the levels of TMPRSS2 (Fig. 3A). Analysis of the effect of extract #7 and its components showed that all components, except CBD, PTWT1.2, and PTWT3.1, profoundly downregulated ACE2 in BJ-5ta cells (Fig. 3B). The effects of PTWT2.2, PTWT6, and PTWT7 were more pronounced than those of the whole extract. Moreover, CBD, CBN, THC, PTWT1.2, and PTWT2.2 significantly decreased the levels of TMPRSS2, while the other compounds and extract #7 increased its expression (Fig. 3B).

We then studied the combined effect between CBD and two selected terpenes: PTWT2.2 and PTWT1.2. WI-38 cells were treated with either 20 μM CBD or the indicated concentration of either PTWT2.2 or PTWT1.2 individually or in combination. Western blot analysis showed a decrease in ACE2 and TMPRSS2 expression in WI-38 cells in response to CBD and PTWT2.2, both individually and in combination (Fig. 3C). Importantly, CBD combined with PTWT2.2 displayed an additive effect on the downregulation of ACE2 and TMPRSS2 expression (Fig. 3C). In contrast, CBD and PTWT1.2 did not show an additive effect (Fig. 3D), although they reduced the expression of ACE2 and/or TMPRSS2 at certain concentrations.

Taken together, these results suggest that CBD and extracts #1, #5, #7, and #129 inhibit the RelA/p65 pathway and modulate the expression of ACE2 and TMPRSS2 both transcriptionally and/or post-transcriptionally. Furthermore, the results indicate that CBD and extracts #1, #5, #7, and #129 activate the AKT pathway. Blockade of the AKT pathway attenuates extract-triggered induction of miR-200c-3p and let-7a-5p, which directly target ACE2 and TMPRSS2, supporting the role of this pathway in the post-transcriptional regulation of ACE2 and TMPRSS2 expression. The results imply that CBD and PTWT2.2 inhibit the expression of ACE2 and TMPRSS2 and have additive effects when combined.

### Cannabis extracts inhibit TNFα/IFNγ-induced IL-6 and IL-8 expression both transcriptionally and post-transcriptionally

**Fig. 4:**
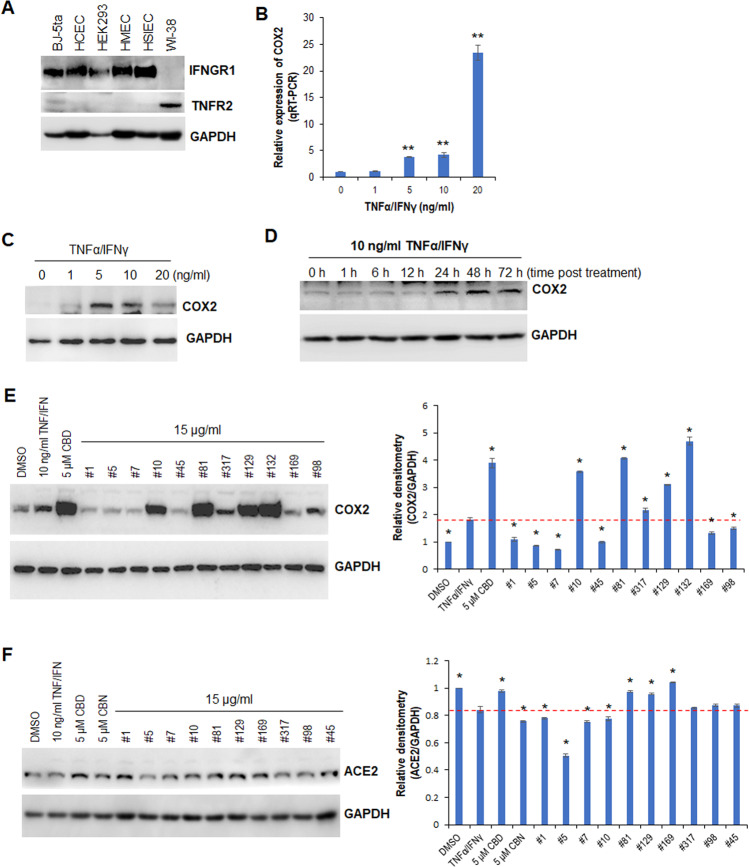
CBD and selected cannabis extracts modulate the expression of COX2 triggered by TNFα/IFNγ. **A** Western blot analysis of IFNGR1 and TNGR2 in the indicated cell lines. **B** qRT-PCR analysis of COX2 in WI-38 cells treated with TNFα/IFNγ for 24 h. **C** Western blot analysis of COX2 in WI-38 cells treated with TNFα/IFNγ for 24 h. **D** Western blot analysis of COX2 in WI-38 cells treated with TNFα/IFNγ for the indicated time points. **E**, **F** Western blot analysis of COX2 (**E**) and ACE2 (**F**) in WI-38 cells treated with either TNFα/IFNγ alone or in combination with the indicated cannabinoids or cannabis extracts; relative densitometry was measured using ImageJ. * indicates *p* < 0.05; ** indicates *p* < 0.01.

The TNFα/IFNγ-induced inflammatory cell model has been widely used in anti-inflammatory drug screening and mechanism studies [20–22]. Recently, TNFα and IFNγ have been shown to synergistically trigger inflammatory cell death that may contribute to the mortality of SARS-CoV-2 infection and cytokine release syndrome [23]. Our recent studies have suggested that cannabis extracts may inhibit the TNFα/IFNγ-induced inflammatory cytokine expression implicated in COVID-19 [20]. Since WI-38 cells only express TNFα receptor 2 (TNFR2; Fig. 4A), we used this cell line as a model system to investigate the anti-inflammatory mechanism of selected cannabis extracts. To establish an inflammatory cell model system, we treated WI-38 cells with TNFα/IFNγ and monitored the induction of COX2, a proinflammatory biomarker, using qRT-PCR and Western blot analysis. TNFα/IFNγ induced COX2 expression in WI-38 cells at both the mRNA and protein levels in a dose- and time-dependent manner (Fig. 4B–D). Because 10 ng/ml TNFα/IFNγ induced COX2 expression at 48 h, we used this dose and time point as a model system to screen anti-inflammatory cannabis extracts. Western blotting showed that the extracts differentially modulated TNFα/IFNγ-induced COX2 expression (Fig. 4E). Extracts #1, #5, #7, #45, #98, and #169 significantly attenuated TNFα/IFNγ-induced COX2 expression (Fig. 4E). Since ACE2 is a crucial SARS-CoV-2 receptor, displayed anti-inflammatory activity, and improved lung function [24], we also determined how TNFα/IFNγ, cannabinoids, and cannabis extracts influence ACE2 expression. Western blot analysis showed that TNFα/IFNγ mildly reduced the ACE2 protein level in WI-38 cells. The level was completely restored by CBD and extracts #81, #129, and #169, and the level was slightly lowered by CBN and extracts #1, #7, and #10 (Fig. 4F). Extract #5 remarkably downregulated ACE2 expression (Fig. 4F).

**Fig. 5:**
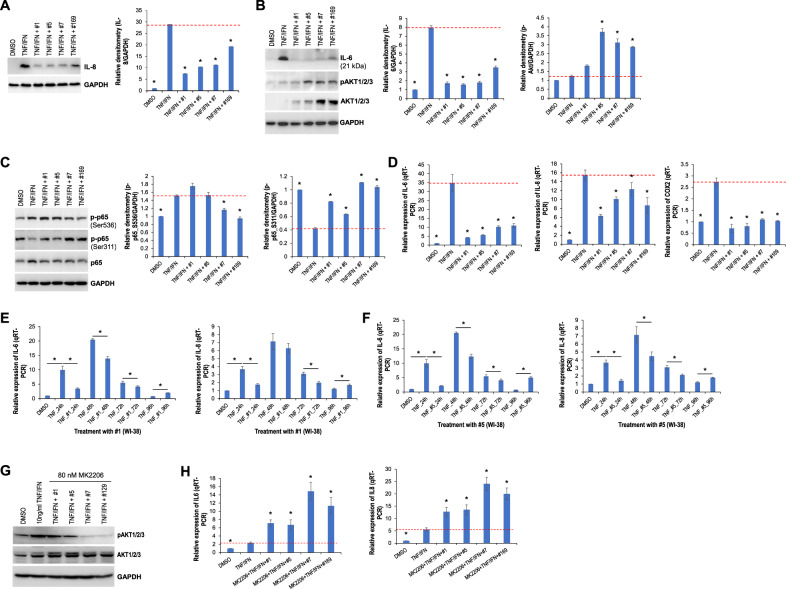
The selected cannabis extracts regulate the expression of proinflammatory cytokines triggered by TNFα/IFNγ and affect signaling pathways. **A**–**C** Western blot analysis of the indicated proteins in WI-38 cells treated with either TNFα/IFNγ alone or in combination with the indicated extracts; relative densitometry was measured using ImageJ. **D** qRT-PCR analysis of IL-6, IL-8, and COX2 in WI-38 cells treated with either TNFα/IFNγ alone or in combination with the indicated extracts. **E**, **F** qRT-PCR analysis of IL-6 and IL-8 in WI-38 cells treated with either TNFα/IFNγ alone or in combination with extract #1 or #5 for the indicated times. **G** Western blot analysis of pAKT/1/2/3 and AKT1/2/3 in WI-38 cells treated with MK2206 for 2 h prior to the addition of either TNFα/IFNγ or in combination with the indicated extracts. **H** qRT-PCR analysis of IL-6 and IL-8 in WI-38 cells treated with MK2206 for 2 h prior to the addition of either TNFα/IFNγ or in combination with the indicated extracts. * indicates *p* < 0.05.

Cannabis extracts #1, #5, #7, and #169 were selected for further analysis because of their capacity to inhibit COX2 expression. We treated WI-38 cells with TNFα/IFNγ in combination with extract #1, #5, #7, or #169, and examined the effect on IL-6, IL-8, and the signaling pathways involved. Western blotting showed that TNFα/IFNγ significantly induced IL-6 and IL-8 expression, and this induction was suppressed by the selected extracts (Fig. 5A, B). TNFα/IFNγ had no effect on the p-pAKT1/2/3 level, whereas the selected extracts increased the p-pAKT1/2/3 level (Fig. 5B). The phosphorylation of p65 at Ser536 was induced by TNFα/IFNγ, and this induction was suppressed by extracts #7 and #169 (Fig. 5C), while extracts #1 and #5 had no effect. Notably, TNFα/IFNγ decreased the level of phosphorylation of p65 at Ser311, and this was restored by the selected extracts (Fig. 5C).

We also looked at the effect of the selected extracts on the transcription of the genes that encode IL-6 and IL-8 to explore the mechanism underlying the cannabis extract-mediated modulation of IL-6 and IL-8. QRT-PCR analysis showed that TNFα/IFNγ elevated the transcription of *IL-6* and *IL-8* in WI-38 cells and that it was weakened by the indicated extracts (Fig. 5D). As expected, TNFα/IFNγ significantly triggered *COX2* transcription, which was profoundly constricted by extracts #1, #5, #7, and #169 (Fig. 5D). The time-dependent effect of selected extracts was examined by treating WI-38 cells with TNFα/IFNγ alone or in combination with extracts #1 and #5 for the indicated times. QRT-PCR analysis indicated a time-dependent induction of TNFα/IFNγ-mediated IL-6 and IL-8, with a transient peak induced 48 h after treatment (Fig. 5E, F). Noticeably, extracts #1 and #5 attenuated the induction of IL-6 and IL-8 in WI-38 cells (Fig. 5E, F), except at the 96-h time point.

**Fig. 6:**
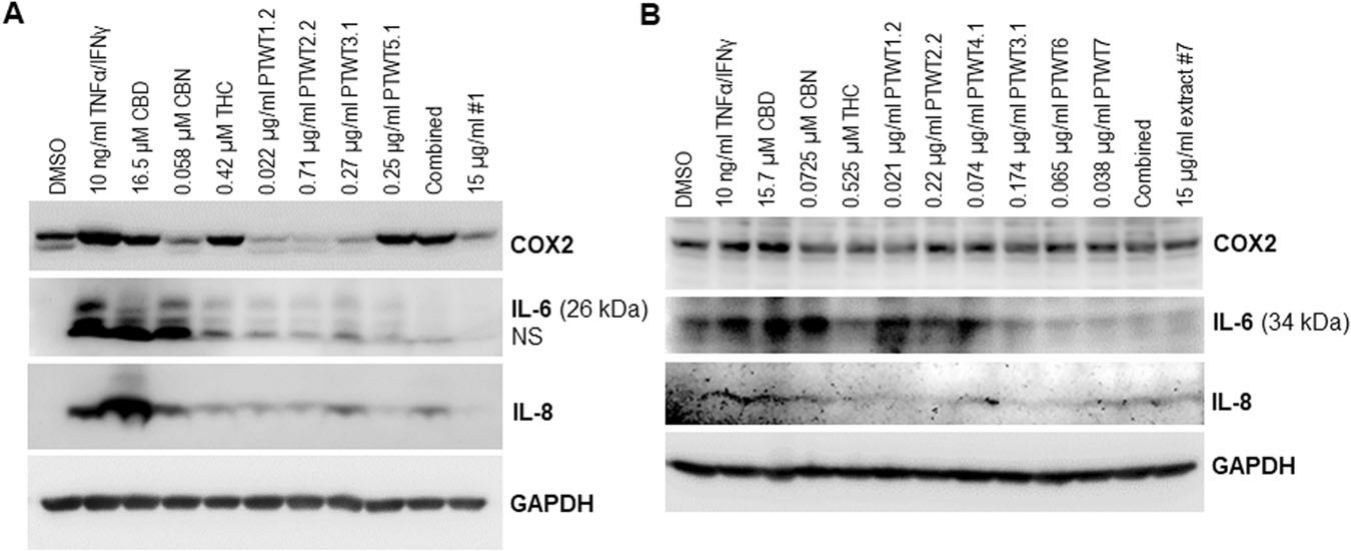
The most abundant compounds of extracts #1 and #7 modulate the expression of inflammatory mediators COX2, IL-6, and IL-8 triggered by TNFα/IFNγ. **A** Western blot analysis of COX2, IL-6 and IL-8 in WI-38 cells treated with either TNFα/IFNγ or in combination with extract #1 or the indicated compounds of extract #1 with the same concentration in the extract. **B** Western blot analysis of COX2, IL-6 and IL-8 in WI-38 cells treated with either TNFα/IFNγ or in combination with extract #7 or the indicated compounds of extract #7 with the same concentration in the extract.

Since the extract-caused suppression of IL-6 and IL-8 induction triggered by TNFα/IFNγ was found to be negatively correlated with the phosphorylation of AKT1/2/3 (Fig. 5B, D), we then looked at the role of the extract-activated AKT pathway in *IL-6* and *IL-8* transcription. For this, the potent AKT1/2/3 inhibitor MK2206 was used. The qRT-PCR results showed that inhibition of the AKT pathway blocked the extract-mediated suppression of IL-6 and IL-8 induction, which led to the transactivation of IL-6 and IL-8 (Fig. 5G, H). To further confirm our findings, AKT1 CRISPR/Cas9 KO plasmid was used to knockdown AKT1 expression. The qRT-PCR indicated that loss-of-function of AKT1 profoundly blocked the inhibition of IL-6 and IL-8 induced by extract #7 (Figure S5), which supported our findings. Although the AKT1 levels reduced by CRISPR KO are still higher than expected, it was sufficient to rescue the TNFα/IFNγ-induced IL-6 and IL-8 transcription/expression via inhibition of AKT1 phosphorylation. In the future, these findings may be further explored in context of stable KO cell lines.Next, we pondered what the most effective anti-inflammatory molecules were in extracts #1 and #7. To investigate this, we exposed WI-38 cells to extracts #1 or #7 or to the same concentrations of the most abundant individual compounds found in these extracts, and monitored COX2, IL-6, and IL-8 expression. Western blot analysis showed that CBD, CBN, THC, and the selected terpenes differentially modulated the induction of COX2, IL-6, and IL-8 triggered by TNFα/IFNγ (Fig. 6A, B). THC markedly downregulated IL-6 and IL-8 induction. CBD and CBN reduced the induction of IL-8 and mature IL-6, while promoting the induction of immature IL-6 (Fig. 6A, B). Most of the selected terpenes attenuated the induction of IL-6 and IL-8. Interestingly, of the combined mixtures that included CBD, THC, CBN, and selected terpenes, extracts #1 and #7 were more effective in downregulating IL-6 expression than the individual compounds (Fig. 6A, B). Collectively, these results suggest that cannabis extracts #1, #5, #7, and #169 attenuate the expression of inflammatory mediators COX2, IL-6, and IL-8 via activation of the AKT pathway; the most abundant compounds in extracts #1 and #7 suppress their expression either individually and/or when combined.

**Fig. 7:**
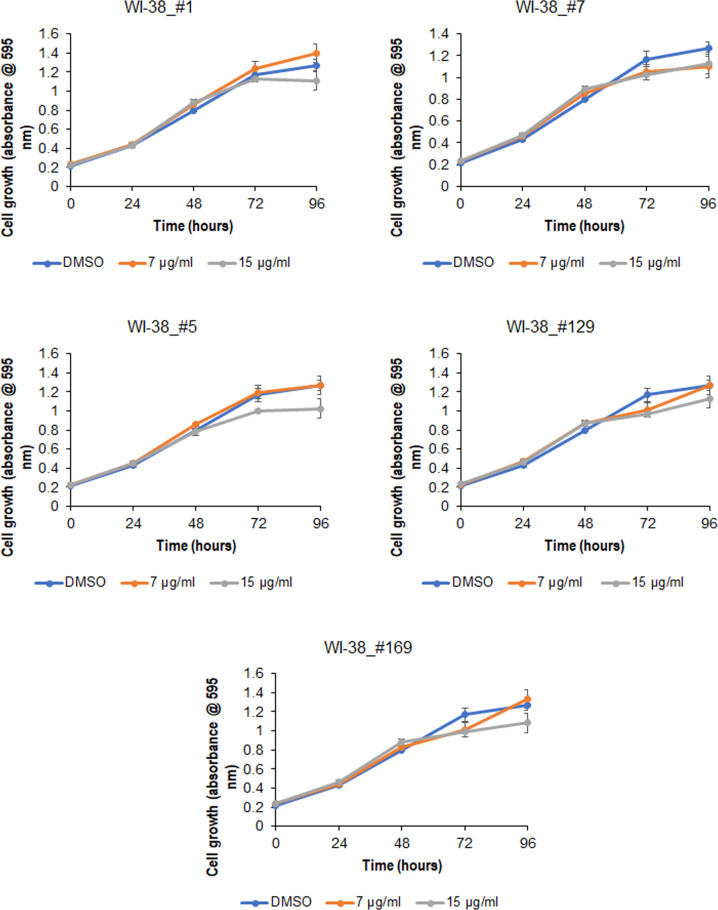
Effect of the selected cannabis extracts on the proliferation of human lung fibroblasts. MTT assay was performed in WI-38 cells treated with the indicated extracts, 0.025% DMSO served as a control.

Selected cannabis extracts did not affect proliferation and growth of normal cells (Fig. 7; Fig. S6).

## Discussion

Here, we evaluated the anti-COVID-19 potential of CBD and high-CBD cannabis extracts and analyzed their effects on ACE2 receptor, which is important for SARS-CoV-2 entry into the cells, along with the anti-inflammatory effects of the extracts. We noted that extracts #1, #5, #7, and #129 were the most potent ACE2 inhibitors, and extracts #1, #5, #7, and #169 had the most profound effects on the inflammation markers. We further investigated the mechanisms of the observed effects.

For the first time, to our knowledge, we have demonstrated that CBD and/or unique high-CBD/low-THC cannabis extracts downregulate ACE2 and TMPRSS2 proteins through miR-200c-3p- and let-7a-5p-targeting via the AKT pathway. The transcriptional induction of miR-200c-3p and let-7a-5p can be blocked by the AKT1/2/3 inhibitor MK2206. It is well known that ACE2 is a key regulator of the renin-angiotensin system (RAS), which plays a crucial role in the maintenance of cardiovascular functions. However, ACE2 can act beyond RAS and has been linked to inflammation and fibrosis [25, 26]. Importantly, ACE2 and TMPRSS2 function as contact points that facilitate SARS-CoV-2 infection [11, 12]. Therefore, as a preventive strategy for COVID-19, there is an emerging need to develop drugs that target ACE2 and/or TMPRSS2. Our recent studies have identified high-CBD *C. sativa* extracts that regulate ACE2 expression in COVID-19 gateway tissues [19]. However, the underlying mechanism(s) has not yet been elucidated.

Here, we screened several cannabis extracts and found that CBD and cannabis extracts **#1, #5, #7, and #129** triggered the expression of miR-200c-3p and let-7a-5p that directly target ACE2 and TMPRSS2, respectively, resulting in a decrease in ACE2 and TMPRSS2 levels. This finding highlights a key role for these two miRNAs in CBD and/or the selected cannabis extract-mediated prevention of COVID-19. This is the first report showing that ACE2 is a direct target of miR-200c-3p; interestingly, miR-200c-3p could also regulate the expression of proinflammatory chemokine IL-8 [27]. Despite the preventive benefit of targeting ACE2 expression in COVID-19 gateway tissues, SARS-CoV spike protein-induced downregulation of ACE2 expression may consequently play a role in acute lung failure, because ACE2 could protect mice from severe acute lung injury [28, 29]. We also noted that knockdown of miR-200c-3p and let-7a-5p failed to block some extract-mediated suppression of ACE2 and TMPRSS2 (Fig. S3). Even though, we can not exclude the contributing role of these two miRNAs in the extract-mediated inhibition of ACE2 and TMPRSS2.

Although several lines of evidence point to cannabis extracts having suppressive effects on ACE2 expression [19, 30], the impact of a single compound found in the extracts on ACE2 expression remains unknown. We have shown here that the most abundant compounds of extracts #1 and #7 differentially modulate ACE2 and TMPRSS2 expression both individually and in combination in a cell type-dependent manner (Fig. 3A, B). Importantly, 20 μM CBD and different concentrations of PTWT2.2 markedly weakened the expression of ACE2 and TMPRSS2 in WI-38 cells and exhibited an additive effect on ACE2 expression (Fig. 3C). In addition to the direct role of terpenes in regulating the expression of ACE2, terpenes may function as SARS-CoV-2 spike protein receptor-binding domain inhibitors, effectively blocking the virus’s interaction with ACE2 [31]. Interestingly, we also noted that the ACE2 antibody used in this study can recognize both glycosylated and non-glycosylated ACE2 (Fig. S7), supporting previous report [32], and may suggest a role of glycosylation in ACE2 functional modulation, albeit evidence has shown that both glycosylated and non-glycosylated ACE2 play a contributing role in SARS-CoV-2 entry [33].

Cytokines and the subset of chemokines have crucial roles in the immune response. However, if this response is too strong, it may lead to a hyper-production of cytokines and chemokines—the cytokine storm—resulting in multi-organ damage. This has been strongly associated with the severity and mortality of COVID-19 [1, 3–9]. Numerous cytokines and chemokines are profoundly elevated in patients with severe COVID-19, including cytokine IL-6, TNFα, and chemokine IL-8 [10, 34–36]. Therefore, inhibitors of IL-6 and its receptor have been proposed as interventions for patients with COVID-19. Several inhibitors that target either IL-6 or its receptor are commercially available, including clazakizumab, sarilumab, siltuximab, sirukumab, and tocilizumab [37]. Application of tocilizumab to patients with severe COVID-19 decreased oxygen requirements (75%) and led to clinical improvements (100%) [38]. In another study tocilizumab treatment reduced the overall requirement for ventilator support, but it did not improve the overall survival [39].

Here, we showed that extracts **#1, #5, #7, and #169** reduced TNFα/IFNγ-triggered induction of inflammatory mediators COX2, IL-6, and IL-8, both transcriptionally and translationally. The effect was mediated by activation of the AKT pathway. This result supports our previous findings that extracts inhibited the expression of COX2, IL-6, and IL-8 in 3D tissue models [20]. Importantly, these extracts did not influence the proliferation of normal fibroblasts. We also noted that the most abundant compounds in extracts #1 and #7 modulated the expression of COX2, IL-6, and IL-8 triggered by TNFα/IFNγ both individually and collaboratively.

Due to their capacity to immunosuppress and downregulate the expression of cytokines and chemokines, cannabinoids have been proposed as anti-inflammatory drugs [40]. There is growing evidence that THC and CBD reduce the production of COX2 [41, 42], IL-1β, IFNγ, IL-6, and TNFα [43–45], which aligns with our findings. A recent elegant study showed that CBD downregulated IL-6 and improved the clinical symptoms of ARDS in a mouse model [45], suggesting that CBD could be an intervention for COVID-19.

Numerous terpenes, including α-bisabolol [46], β-caryophyllene [47, 48], geraniol [49], and valencene [50], have been shown to attenuate the production/release of IL-1β, IL-6, and TNFα via blockade of the NF-κB pathway, both in vitro and in vivo. The NF-κB pathway is one of the most well-defined key pathways that triggers immune and inflammatory responses [51, 52]. Members of the transcription factor NF-κB family, including RelA (p65), RelB, c-Rel, p50, and p52, play crucial roles in many biological and pathological processes by controlling the transcription of NF-κB target genes. Post-translational modifications can modulate NF-κB’s activity. Phosphorylation of p65 at Ser536 directly transactivates the expression of proinflammatory cytokines and chemokines [53], while phosphorylation at Ser311 may primarily contribute to the nuclear translocation of p65 [54]. Here, we found a differential impact of extracts #1, #5, #7, and #169 on the phosphorylation of p65 at Ser311 and Ser536. Although extracts #7 and #169 induced phosphorylation at Ser311, they significantly attenuated TNFα/IFNγ-induced phosphorylation at Ser536 (Fig. 5C). That activity may contribute to the transcriptional inhibition of IL-6, IL-8, and COX2 mediated by these two extracts.

The PI3K/AKT pathway has a role in cell proliferation, cell survival, the cell cycle, apoptosis, glycogen metabolism, and inflammation. The AKT pathway is known to play a pivotal role in the production of cytokines [55, 56].

We show here that extracts **#**1, #5, #7, and #169 upregulated the levels of phosphorylated AKT1/2/3 that were inversely correlated with transcription of IL-6, IL-8, and COX2 (Fig. 5B, D). The extract-mediated transcriptional suppression was completely abolished by blockade of AKT activation (Fig. 5G, H), suggesting a suppressive role of this pathway in the transcription of inflammatory factors. Our results disagree with previous findings that showed the AKT pathway contributes to the release of inflammatory factors. However, several lines of evidence have also indicated an inhibitory impact of AKT activation on the expression of proinflammatory cytokines [57, 58], supporting our results. We also revealed a key role of AKT-dependent expression of miR-200c-3p and let-7a-5p in the post-transcriptional silencing of ACE2 and TMPRSS2, which may contribute to CBD and/or cannabis extract-mediated downregulation of the SARS-CoV-2 receptor proteins. Post-transcriptional regulation of SARS-CoV-2 receptors ACE2 and TMPRSS2 has not yet drawn much attention, although *in silico* analysis recently identified several miRNAs potentially targeting either ACE2 or TMPRSS2 [59], including miR-200c-3p and let-7a-5p. Further studies are needed to define the roles of non-coding RNAs in the molecular etiology of COVID-19.

In conclusion, our high-CBD/low-THC cannabis extracts suppress the expression of SARS-CoV-2 host entry proteins ACE2 and TMPRSS2 and the induction of inflammatory mediators COX2, IL-6, and IL-8 via the AKT pathway, transcriptionally and/or post-transcriptionally. Together, these results highlight the potential anti-COVID-19 features of the extracts, supporting the urgent need for clinical trials for these unique extracts. It should be noted, however, that not all extracts, despite being CBD-dominant, were effective, indicating that there are some specific modulating components beyond those terpenes that were analyzed in our work. The consistency of the concentration of individual ingredients in the presented extracts is of potential concern since slight changes in the growth conditions may alter the total amounts of individual ingredients and their ratios. It would be therefore important to identify the proper ratios of combination of single ingredients to come up with ideal formulation for future potential clinical studies Such approach would allow to avoid unpredictability associated with growing cannabis and using whole flower extracts for future clinical use.

## Materials and Methods

### Cannabis plant growth and extract preparation

The information regarding cannabis plant growth and extract preparation has been detailed previously [19].

### Cell culture

Human normal foreskin fibroblasts (BJ-5ta), purchased from American Type Culture Collection (ATCC, Manassas, USA), were cultured in Dulbecco’s Modified Eagle’s Medium supplemented with 10% fetal bovine serum (FBS). Human primary colonic epithelial cells (HCEC), purchased from Cell Biologics (Chicago, USA), were cultured in Epithelial Cell Medium /w Kit. Human embryonic kidney epithelial cells (HEK293), purchased from ATCC, were cultured in Eagle’s Minimum Essential Medium supplemented with 10% FBS. Human mammary epithelial cells (HMEC), purchased from Invitrogen were cultured in a HuMEC basal serum-free medium (Invitrogen) containing HuMEC supplement (Invitrogen). Human primary small intestinal epithelial cells (HSIEC), purchased from Cell Biologics were cultured in Epithelial Cell Medium /w Kit. Human lung fibroblasts (WI-38), purchased from ATCC, were cultured in Eagle’s Minimum Essential Medium supplemented with 10% FBS. All cell lines were incubated at 37 °C in a humidified atmosphere of 5% CO_2_. Mycoplasma contamination was regularly monitored using a Mycoplasma PCR Detection kit (Applied Biological Materials Inc., Richmond, BC, Canada) and eradicated using BM-Cyclin (Sigma-Aldrich, Darmstadt, Germany) according to the manufacturer’s instructions.

### Treatment with cannabinoids and cannabis extracts

BJ-5ta and WI-38 cells grown to 80% confluency were treated with either 5 μM, 10 μM, or 15 μM CBD, as well as CBN or THC for 24 h; 0.47% methanol served as a control. BJ-5ta and WI-38 cells grown to 80% confluency were treated with either 10 μM CBD or 15 μg/ml cannabis extracts, including #1, #5, #7, #10, #45, #81, #317, #98, #129, #132, and #169 for 24 h; 0.025% DMSO served as a control. BJ-5ta and WI-38 cells grown to 80% confluency were treated with either 7 μg/ml, 10 μg/ml, or 15 μg/ml cannabis extracts #1 or #5 for 24 h; 0.025% DMSO served as a control.

### Bioinformatic analysis

MicroRNAs (miRNAs/miRs) that target either ACE2 or TMPRSS2 were predicted using TargetScanHuman 7.2 software (http://www.targetscan.org/vert_72/).

### miRNA quantitative real-time RT-PCR (qRT-PCR)

WI-38 cells grown to 80% confluency were treated with either 10 μM CBD or 15 μg/ml cannabis extracts #1, #5, #7, and #129; 0.025% DMSO served as a control. Twenty-four hours after treatment, total RNA was isolated using TRIzol RNA isolation reagent (ThermoFisher Scientific) and subjected to qRT-PCR analysis miScript II RT kit (QIAGEN) and QuantiTect SYBR Green PCR Master mix (QIAGEN) using specific primers to either hsa-miR-200b-3p, hsa-miR-200c-3p, hsa-let-7a-5p, or hsa-let-7b-5p, according to the manufacturers’ instructions. Human RNU6-2 (QIAGEN) served as a loading control. All qRT-PCR experiments were performed in triplicate, and the data were analyzed using the comparative Ct method. The results were shown as a fold induction of the genes indicated.

### Western blot analysis

The indicated cells were rinsed twice with ice-cold PBS and scraped off the plate in a RIPA buffer; 30-100 μg of protein per sample was electrophoresed on 8%, 10%, or 12% SDS-PAGE and electrophoretically transferred to a PVDF membrane (Amersham Hybond™-P, GE Healthcare, Illinois, USA) at 4 °C for 1.5 h. Blots were incubated for 1 h with 5% non-fat dry milk to block nonspecific binding sites and subsequently incubated at 4 °C overnight with 1:200 to 1:1,000 dilution of polyclonal/monoclonal antibodies against ACE2 (Cat# ab15348), cannabinoid receptor 1 (CB1, Cat# ab259323), cannabinoid receptor 2 (CB2, Cat# ab3561), COX2 (Cat# ab15191), IFNGR1 (Cat# ab134070), IL-8 (Cat# ab18672), TMPRSS2 (Cat# ab92323), TNFR2 (Cat# ab109322) (all from Abcam, Cambridge, UK), NF-κB p65 (#8242), phospho-NF-κB p65 (Ser536, Cat# 3033) (from Cell Signaling Technology, Danvers, USA), AKT1 (Cat# sc-377457), AKT1/2/3 (Cat# sc-81434), IL-6 (Cat# sc-130326), p-p65 (Ser311, Cat# sc-33039), pAKT1 (Ser473, Cat# sc-293125), or pAKT1/2/3 (Cat# sc-514032) (all from Santa Cruz Biotechnology, Dallas, USA). Immunoreactivity was detected using a peroxidase-conjugated antibody and visualized by an ECL Plus Western Blotting Detection System (GE Healthcare, Illinois, USA). The blots were stripped before re-probing with antibody against GAPDH (Abcam, Cambridge, UK). Densitometry of bands was measured and normalized with that of GAPDH using ImageJ.

### mRNA qRT-PCR

WI-38 cells grown to 80% confluency were treated with 10 μM CBD or 15 μg/ml extracts #1 or #5 or #7 or #129 for 24 h, DMSO served as a control; or treated with 7 or 15 or 30  μg/ml extracts #1 or #5 for 24 h. WI-38 cells grown to 70% confluency were exposed to either 10 ng/ml TNFα/IFNγ or in combination with 15 μg/ml extracts #1 or #5 or #7 or #169 for 48 h, 0.025% DMSO served as a control; or treated with either 10 ng/ml TNFα/IFNγ or in combination with 15 μg/ml extracts #1 or #5 for the indicated time-course, 0.025% DMSO served as a control. At the indicated time point after treatment, total RNA was isolated using TRIzol RNA isolation reagent (ThermoFisher Scientific) and subjected to qRT-PCR analysis with iScript Select cDNA Synthesis kit (Bio-Rad) and SsoFast Evagreen SYBR Supermix (Bio-Rad) using primers specific for ACE2 or TMPRSS2 or IL-6 or IL-8 or COX2 according to the manufacturer’s instructions. Real-time quantitative RT-PCR analysis was carried out with CFX96 Real-Time System (Bio-Rad). *Glyceraldehyde-3-phosphate dehydrogenase* (GAPDH) was used as the internal control to standardize the expression [60]. All experiments for real-time RT-PCR were performed in triplicate and data was analyzed using the comparative Ct method [61]. Results are shown as fold induction of mRNA.

### Inhibition of AKT pathway and combined treatment

WI-38 cells grown to 80% confluency were treated with 80 nM of well-established AKT inhibitor MK2206 [62–69] (Selleckchem) for 2 h prior to addition of 10 μM CBD or 15 μg/ml cannabis extracts #1 or #5 or #7 or #129. At 24 h after treatment, the whole cellular lysates were prepared and total RNA was isolated and stored at −20 °C.

### Construction of luciferase reporters

ACE2 and TMPRSS2 luciferase reporters bearing either a predicted wild-type or mutant hsa-miR-200b/c-3p or hsa-let-7a/b-5p binding site were generated by synthesizing the following oligos: wtACE2-3′UTR1: 5′-/5Phos/CTAGACATTGACATTGCTTTCAGTATTTATG-3′, wtACE2-3′UTR2: 5′-/5Phos/AATTCATAAATACTGAAAGCAATGTCAATGT-3′; mtACE2-3′UTR1: 5′-/5Phos/CTAGACATTGACATTGCTTTCTTAAAATATG-3′, mtACE2-3′UTR2: 5′-/5Phos/AATTCATATTTTAAGAAAGCAATGTCAATGT-3′, wtTMPRSS2-3′UTR1: 5′-/5Phos/CTAGATGTTTCTACACATTGCTACCTCAG-3′, wtTMPRSS2-3′UTR2: 5’-/5Phos/AATTCTGAGGTAGCAATGTGTAGAAACAT-3′; mtTMPRSS2-3′UTR1: 5’-/5Phos/CTAGATGTTTCTACACATTGGAAATTTTG-3′, and mtTMPRSS2-3′UTR2: 5’-/5Phos/AATTCAAAATTTCCAATGTGTAGAAACAT-3′. After annealing, the double-stranded oligos were cloned downstream of the luciferase gene in the pGL3-Basic vector, between *Xba*I and *EcoR*I (a linker introduced by Mr. James Meservy) to generate Luc-wtACE2-3′UTR, Luc-mtACE2-3′UTR, Luc-wtTMPRSS2-3′-UTR, and Luc-mtTMPRSS2-3′UTR reporters. The sequence identity was confirmed by automated DNA sequencing.

### Luciferase assay

HEK293 cells grown to 80% confluency in six-well plates were transiently cotransfected with 100 ng of either Luc-wtACE2-3′UTR or Luc-mtACE2-3′UTR or Luc-wtTMPRSS2-3′UTR or Luc-mtTMPRSS2-3′UTR luciferase reporter and 5 ng of pRL-TK plasmid in combination with the indicated concentration of hsa-miR-200c-3p or hsa-let-7a-5p mimics using Lipofectamine 3000 (Invitrogen) per manufacturer’s instruction. At 24 h after transfection, the cells were lysed in passive lysis buffer and the relative luciferase activity was determined by the Dual-Luciferase Reporter Assay System (Promega) using a luminometer (FLUOstar Omega, BMG LABTECH) with Firefly luciferase data normalized to Renilla. Two independent assays were repeated, each was done in duplicate.

### Inhibition of miR-200c-3p and let-7a-5p

WI-38 cells grown to 80% confluency were transfected with 50 nM miRCURY LNA miRNA power inhibitor targeting either miR-200c-3p or let-7a-5p (QIAGEN), using lipofectamine 3000 (ThermoFisher Scientific), according to the manufacturer’s instructions; negative control A (QIAGEN) served as a control. At 24 h after transfection, the cells were exposed to either 0.025% DMSO or 10 μM CBD or 15 μg/ml of the indicated extracts. At 24 h after treatment, the cells were harvested, washed once with cold PBS, lysed in RIPA buffer containing cocktail proteinase inhibitors, and stored at −20 °C.

### Treatment with extract main compounds

BJ-5ta and WI-38 cells grown to 80% confluency were exposed to the the indicated concentration of the most abundant compounds of either extract #1 (including CBD, CBN, THC, and terpenes PTWT1.2, PTWT2.2, PTWT3.1, and PTWT5.1) or #7 (including CBD, CBN, THC, and terpenes PTWT1.2, PTWT2.2, PTWT4.1, PTWT3.1, PTWT6, and PTWT7) individually or in combination. To analyze the effects of combinations, WI-38 cells grown to 80% confluency were treated with either 20 μM CBD or the indicated concentration of terpenes individually or in combination. At 24 h after treatment, whole cellular lysates were prepared and stored at −20 °C.

### Inflammation induction

WI-38 cells grown to 80% confluency were treated either with different concentrations of TNF-α/IFN-γ (Sigma) for 24 h or with 10 ng/ml TNF-α/IFN-γ [70] for the indicated time points.

### Screening of anti-inflammatory cannabis extracts

WI-38 cells grown to 80% confluency were treated with 10 ng/ml TNFα/IFNγ alone or in combination with 5 µM CBD or 15 µg/ml cannabis extracts #1, #5, #7, #10, #45, #81, #98, CD10, #129, #132, or #169, 0.025% DMSO served as a control. At 48 h after treatment, cells were washed three times with cold PBS and whole cellular lysates were prepared and stored at −20 °C.

### Knockdown of AKT1 and combined treatment

WI-38 cells grown to 50% confluency were transfected with 1 μg or 3 μg of either Akt1 CRISPR/Cas9 KO or control CRISPR/Cas9 plasmid (QIAGEN) using lipofectamine 3000 (ThermoFisher Scientific) according to manufacturer’s instructions. At 24 h after transfection, the untransfected cells were exposed to either 10 ng/ml TNFα/IFNγ or 0.025% DMSO, while transfected cells were treated with 10 ng/ml TNFα/IFNγ in combination with 15 μg/ml extract #7. At 48 h after treatment, whole cellular lysates were prepared, total RNA was isolated, and stored at −20 °C.

### MTT assay

Once cells grown to 70–90% confluency, 3 × 10^3^ BJ-5ta cells/well, 3 × 10^3^ HSIEC cells/well, and 5 × 10^3^ WI-38 cells/well were replated in 96-well plates. At 24 h after incubation, cells were treated with either 7.5 µg/ml or 15 µg/ml of extracts #1, #5, #7, #129, and/or #169, 0.025% DMSO served as a control. Assays were performed with 3-(4,5-Dimethylthiazol-2-yl)-2,5-diphenyltetrazolium bromide (MTT) using the Cell Proliferation Kit I (Roche Diagnostics GmbH) in triplicate, as described by the manufacturer. The spectrophotometric absorbance of samples was measured at 595 nm using a microtiter plate reader (FLUOstar Omega, BMG LABTECH).

### Statistical analysis

Two-tailed Student’s *t*-test was used to determine the statistical significance of the difference in expression of ACE2, AKT1/2/3, COX2, IL-6, IL-8, let-7a-5p, let-7b-5p, miR-200b-3p, miR-200c-3p, p65, p-p65 (Ser311), p-p65 (Ser536), pAKT1/2/3, and TMPRSS2, cell growth and luciferase activity. *P* < 0.05 was considered significant. Definition of “center values” as mean; definition of error bars as S.D.

## Supplementary information

Supplementary figure legends

Suppl figures - revised

Original images

## Data Availability

All data are available in the main text or the supplementary materials.
