# Supplementary material for: New AKT-dependent mechanisms of anti-COVID-19 action of high-CBD Cannabis sativa extracts
Source: Cell Death Discov. 2022 Mar 11;8:110. doi: 10.1038/s41420-022-00876-y (PMC8913855; doi:10.1038/s41420-022-00876-y)
Supplement: Supplementary file 1 — Supplementary figure legends [file 41420_2022_876_MOESM1_ESM.docx]

**Supplementary figures**

**Figure S1.** **miRNAs potentially targeting ACE2**.

(A) Binding motif between ACE2 3’UTR and miR-200b-3p, miR-200c-3p, and miR-429.

(B) The target sequence of ACE2 3’UTR is highly conserved among species.

**Figure S2. miRNAs potentially targeting TMPRSS2**.

(A) Binding motif between TMPRSS2 3’UTR and let-7 family members, miR-4458, and miR-4500.

(B) The target sequence of TMPRSS2 3’UTR is highly conserved among species.

**Figure S3. miR-200c-3p and let-7a-5p inhibitors block CBD and extracts #1 and #129 induced downregulation of ACE2 and/or TMPRSS2**. (A, B) WI-38 cells were transfected with 50 nM either miR-200c-3p inhibitor (A) or let-7a-5p inhibitor (B) or negative control A; at 24 h after transfection, the cells were exposed to either 0.025% DMSO or 10 μM CBD or 15 μg/ml of the indicated extracts; at 24 h after treatment, whole cellular lysates were prepared and subjected to Western blot analysis using antibodies against ACE2 or TMPRSS2; relative densitometry was measured using ImageJ.

* indicates p<0.01. ** indicates p<0.005.

**Figure S4. CBD and cannabis extracts regulate expression of ACE2 and TMPRSS2 in BJ-5ta cells**.

(A, B**)** Western blot analysis of the indicated proteins in BJ-5ta cells treated with either CBD or the indicated cannabis extracts for 24 h; relative densitometry was measured using ImageJ.

* indicates p<0.05.

**Figure S5. Knockdown of Akt1 blocks extract #7-mediated suppression of IL-6 and IL-8 induction**. (A, B) WI-38 cells were transfected with 1 μg or 3 μg of either Akt1 CRISPR/Cas9 KO or control CRISPR/Cas9 plasmid; at 24 h after transfection, the cells were exposed to either 10 ng/ml TNFα/IFNγ alone or in combination with 15 μg/ml extract #7; at 48 h after treatment, whole cellular lysates were prepared and subjected to Western blot analysis using antibodies to pAkt1 or Akt1; relative densitometry was measured using ImageJ (A); total RNA was isolated and subjected to qRT-PCR using primer set of either IL-6 or IL-8 (B).

* indicates p<0.05. ** indicates p<0.01.

**Figure S6. Effect of the selected cannabis extracts on proliferation of human foreskin fibroblasts**.

MTT assay was performed in BJ-5ta cells treated with the indicated extracts, 1% DMSO served as control.

**Figure S7. Specificity of ACE2 antibody**. Western blot analysis was performed using antibody against ACE2 which was applied in this study. Lane 1: Whole cellular lysate prepared from Hep G2 cells with wild-type ACE2 (Hep G2_ACE2+/+) was purchased from Abcam; lane 2: Whole cellular lysate prepared from Hep G2 cells with ACE2 knockout (Hep G2_ACE2-/-) was purchased from Abcam; lane 3: Whole cellular lysate was prepared from WI-38 cells which were exposed to extract #1 for 24 h; lane 4: Whole cellular lysate was prepared from WI-38 cells which was treated with extract #10 for 24 h.
