# Supplementary material for: New AKT-dependent mechanisms of anti-COVID-19 action of high-CBD Cannabis sativa extracts
Source: Cell Death Discov. 2022 Mar 11;8:110. doi: 10.1038/s41420-022-00876-y (PMC8913855; doi:10.1038/s41420-022-00876-y)
Supplement: Supplementary file 2 — Suppl figures - revised [file 41420_2022_876_MOESM2_ESM.pptx]

## Slide 1
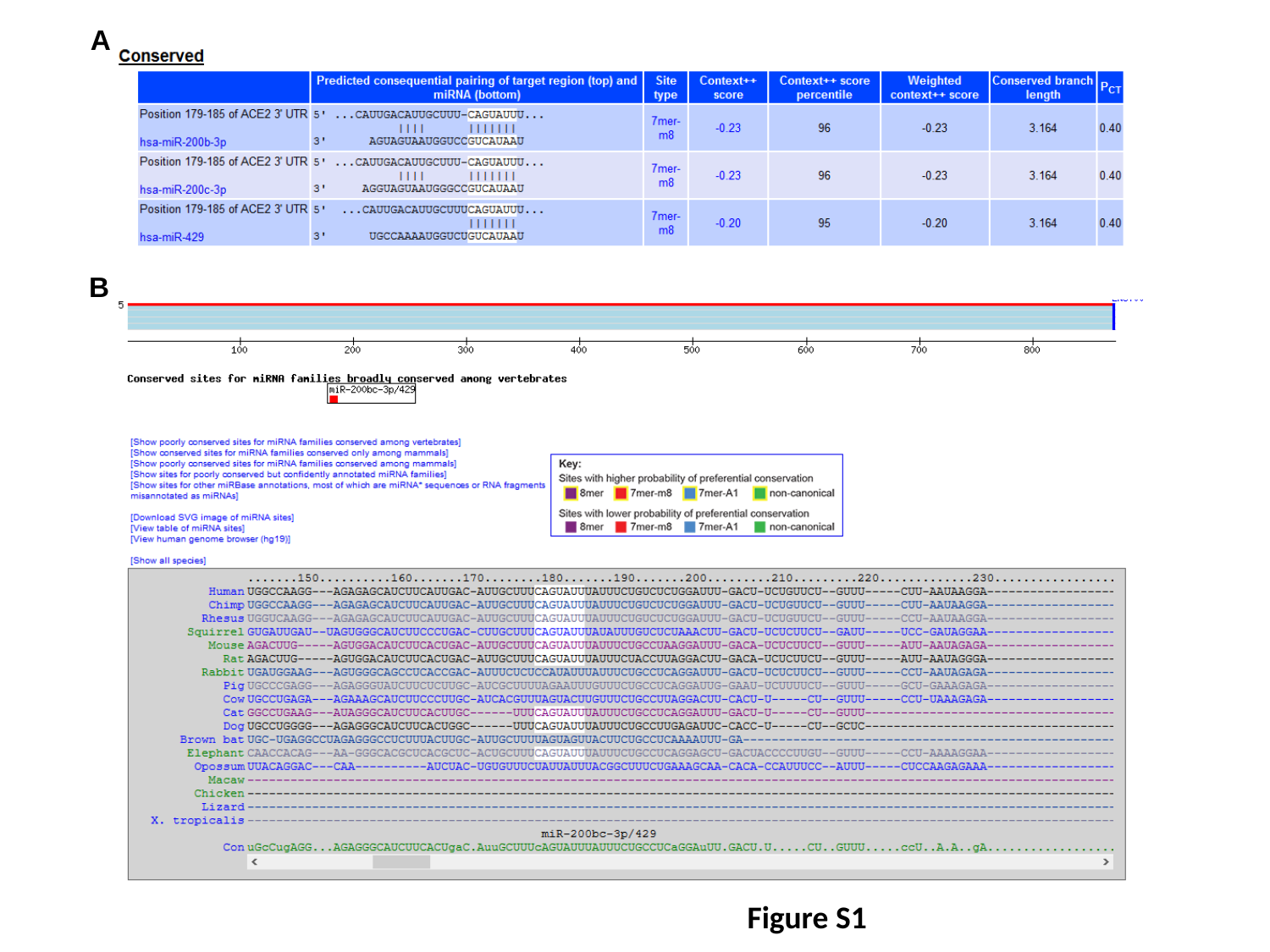

A
B
Figure S1

## Slide 2
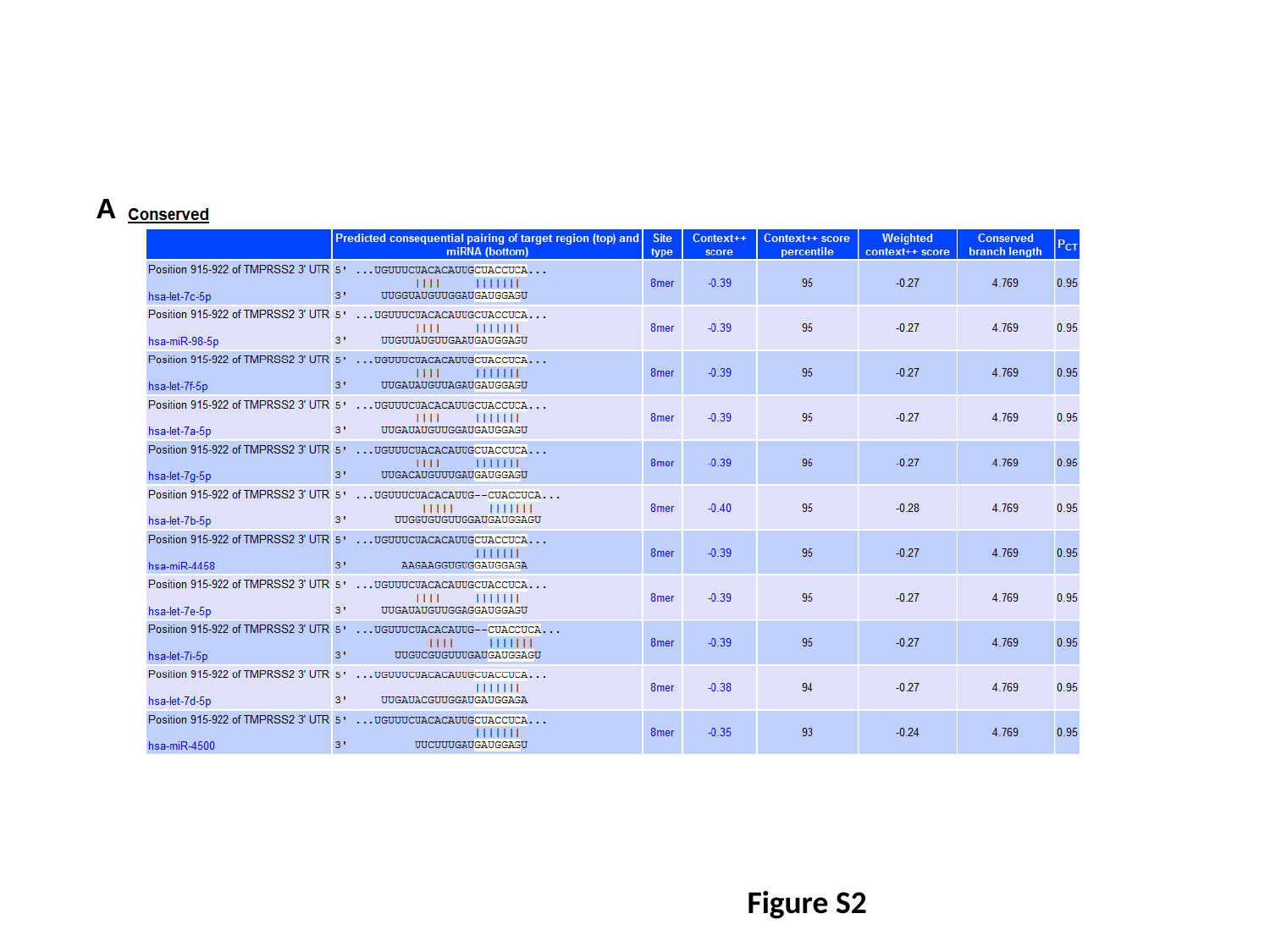

A
Figure S2

## Slide 3
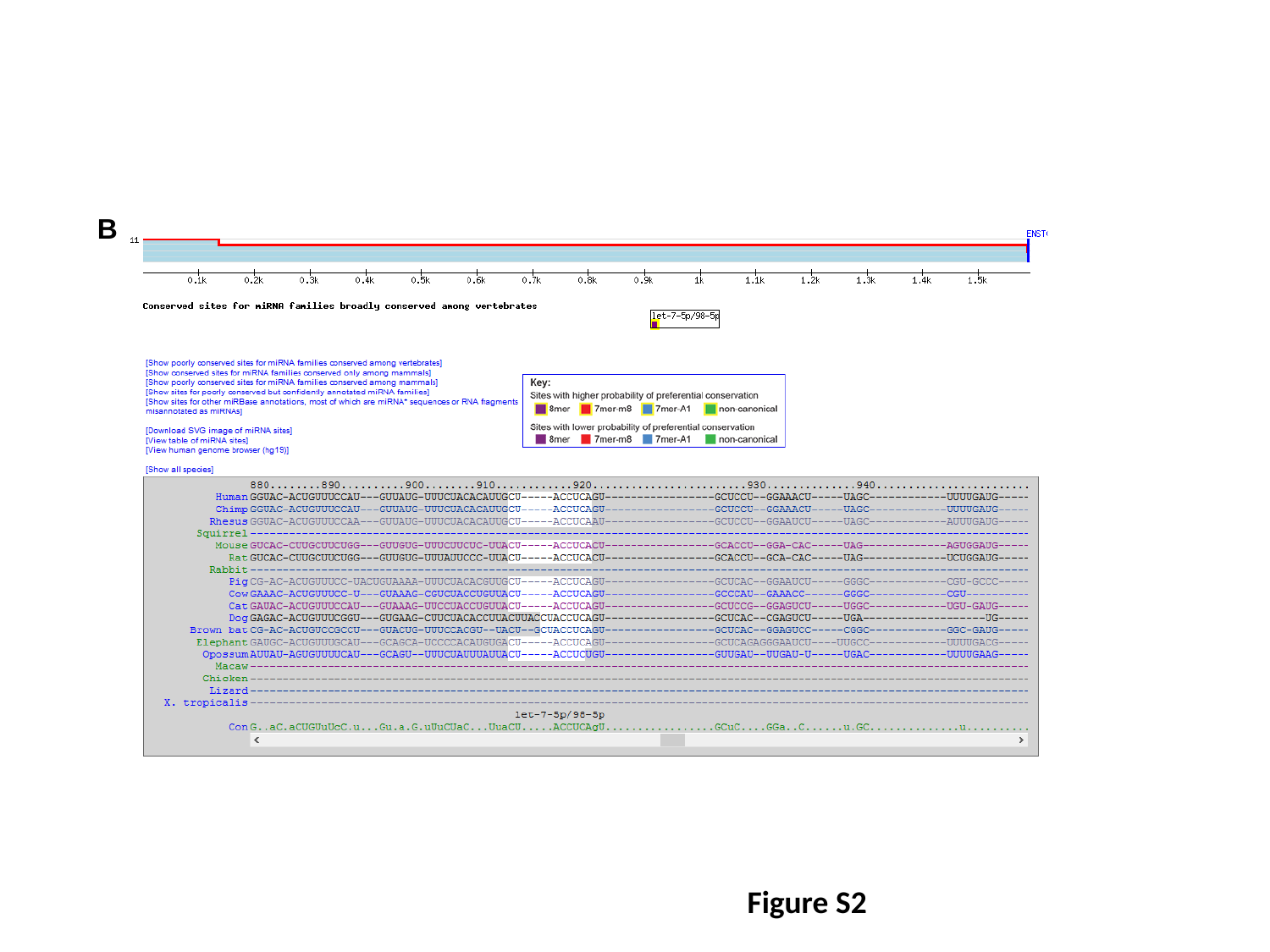

B
Figure S2

## Slide 4
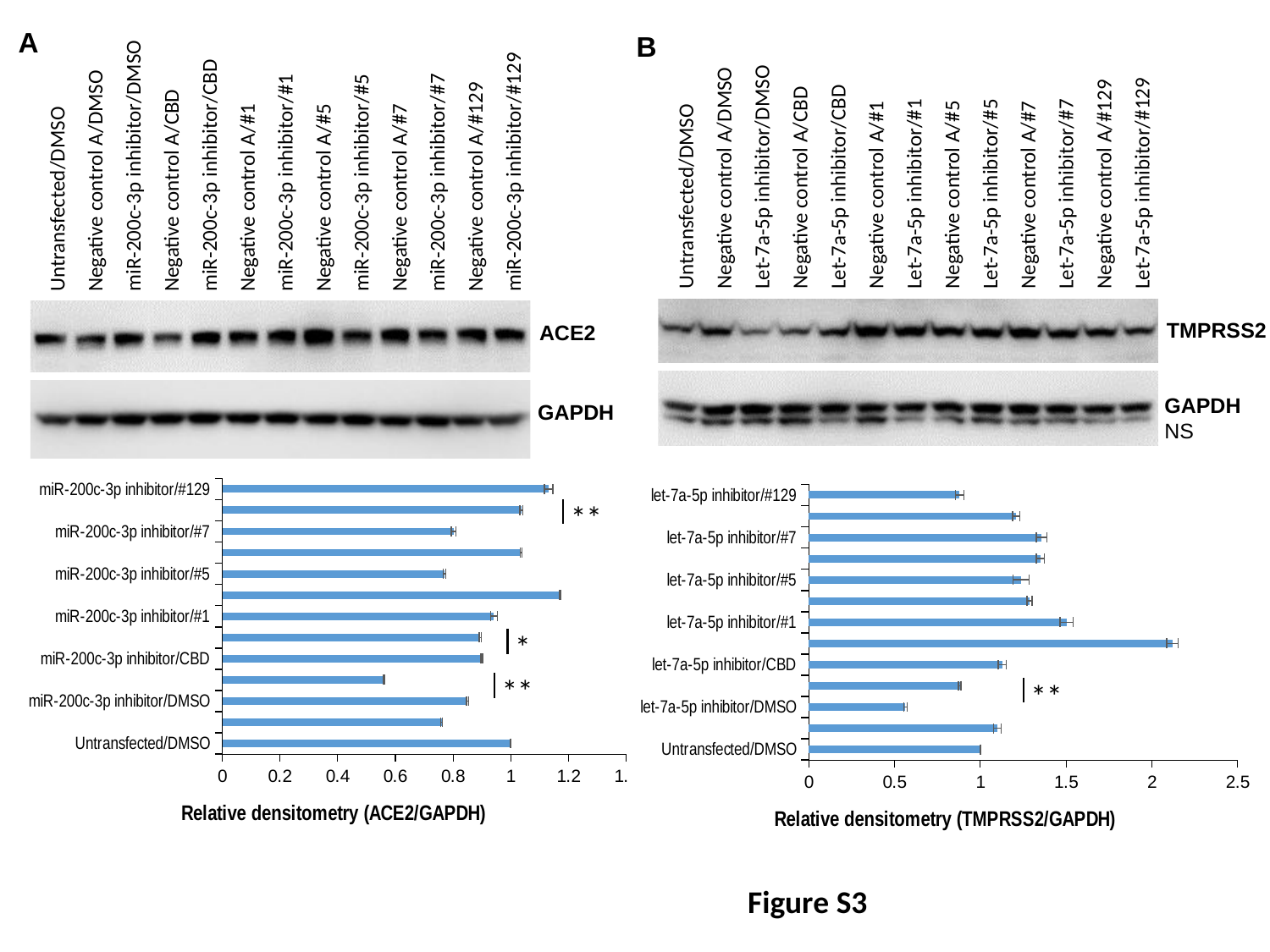

Untransfected/DMSO
Negative control A/DMSO
miR-200c-3p inhibitor/DMSO
Negative control A/CBD
miR-200c-3p inhibitor/CBD
Negative control A/#1
miR-200c-3p inhibitor/#1
Negative control A/#5
miR-200c-3p inhibitor/#5
Negative control A/#7
miR-200c-3p inhibitor/#7
Negative control A/#129
miR-200c-3p inhibitor/#129
Untransfected/DMSO
Negative control A/DMSO
Let-7a-5p inhibitor/DMSO
Negative control A/CBD
Let-7a-5p inhibitor/CBD
Negative control A/#1
Let-7a-5p inhibitor/#1
Negative control A/#5
Let-7a-5p inhibitor/#5
Negative control A/#7
Let-7a-5p inhibitor/#7
Negative control A/#129
Let-7a-5p inhibitor/#129
A
B
TMPRSS2
ACE2
GAPDH
NS
GAPDH
### Chart
| Category | |
|---|---|
| Untransfected/DMSO | 1.0 |
| Negative control A/DMSO | 0.7600305505721797 |
| miR-200c-3p inhibitor/DMSO | 0.8497951775853849 |
| Negative control A/CBD | 0.560635889943168 |
| miR-200c-3p inhibitor/CBD | 0.899875789563283 |
| Negative control A/#1 | 0.8938259121281326 |
| miR-200c-3p inhibitor/#1 | 0.9414595417283612 |
| Negative control A/#5 | 1.1701350443316267 |
| miR-200c-3p inhibitor/#5 | 0.7699499422724786 |
| Negative control A/#7 | 1.035760146383482 |
| miR-200c-3p inhibitor/#7 | 0.8021100536081729 |
| Negative control A/#129 | 1.036667595627567 |
| miR-200c-3p inhibitor/#129 | 1.1309476260405769 |
### Chart
| Category | |
|---|---|
| Untransfected/DMSO | 1.0 |
| Negative control A/DMSO | 1.0982306463634253 |
| let-7a-5p inhibitor/DMSO | 0.5628796173637906 |
| Negative control A/CBD | 0.8795398904507481 |
| let-7a-5p inhibitor/CBD | 1.1274269667624284 |
| Negative control A/#1 | 2.1194870456005113 |
| let-7a-5p inhibitor/#1 | 1.5019782050009853 |
| Negative control A/#5 | 1.2865702628526432 |
| let-7a-5p inhibitor/#5 | 1.2367935844015434 |
| Negative control A/#7 | 1.349714844510263 |
| let-7a-5p inhibitor/#7 | 1.356504627503203 |
| Negative control A/#129 | 1.2090716758406121 |
| let-7a-5p inhibitor/#129 | 0.8791544191069472 |**
*
**
**
Figure S3

## Slide 5
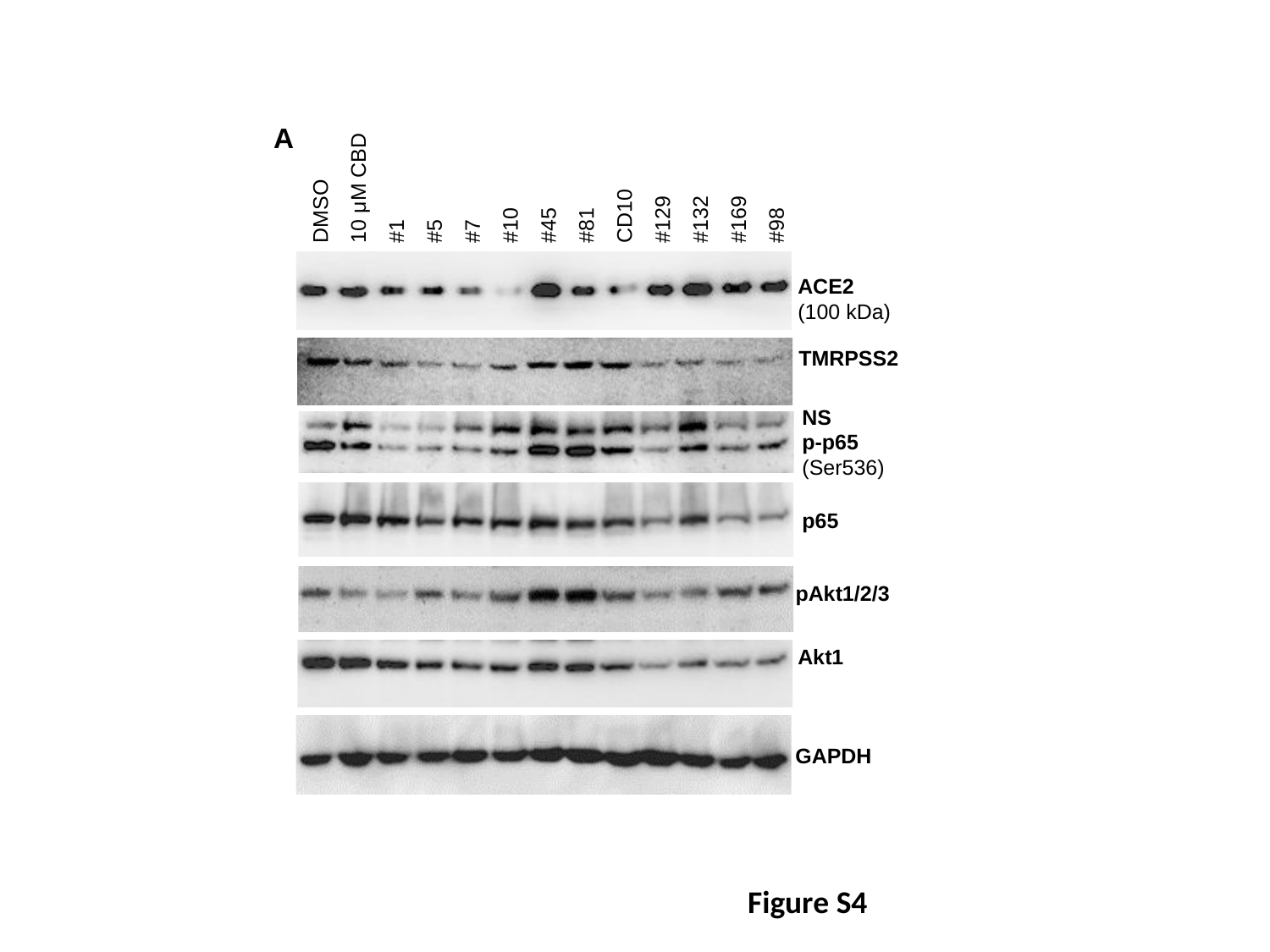

DMSO
10 μM CBD
#1
#5
#7
#10
#45
#81
CD10
#129
#132
#169
#98
A
ACE2
(100 kDa)
TMRPSS2
NS
p-p65
(Ser536)
p65
pAkt1/2/3
Akt1
GAPDH
Figure S4

## Slide 6
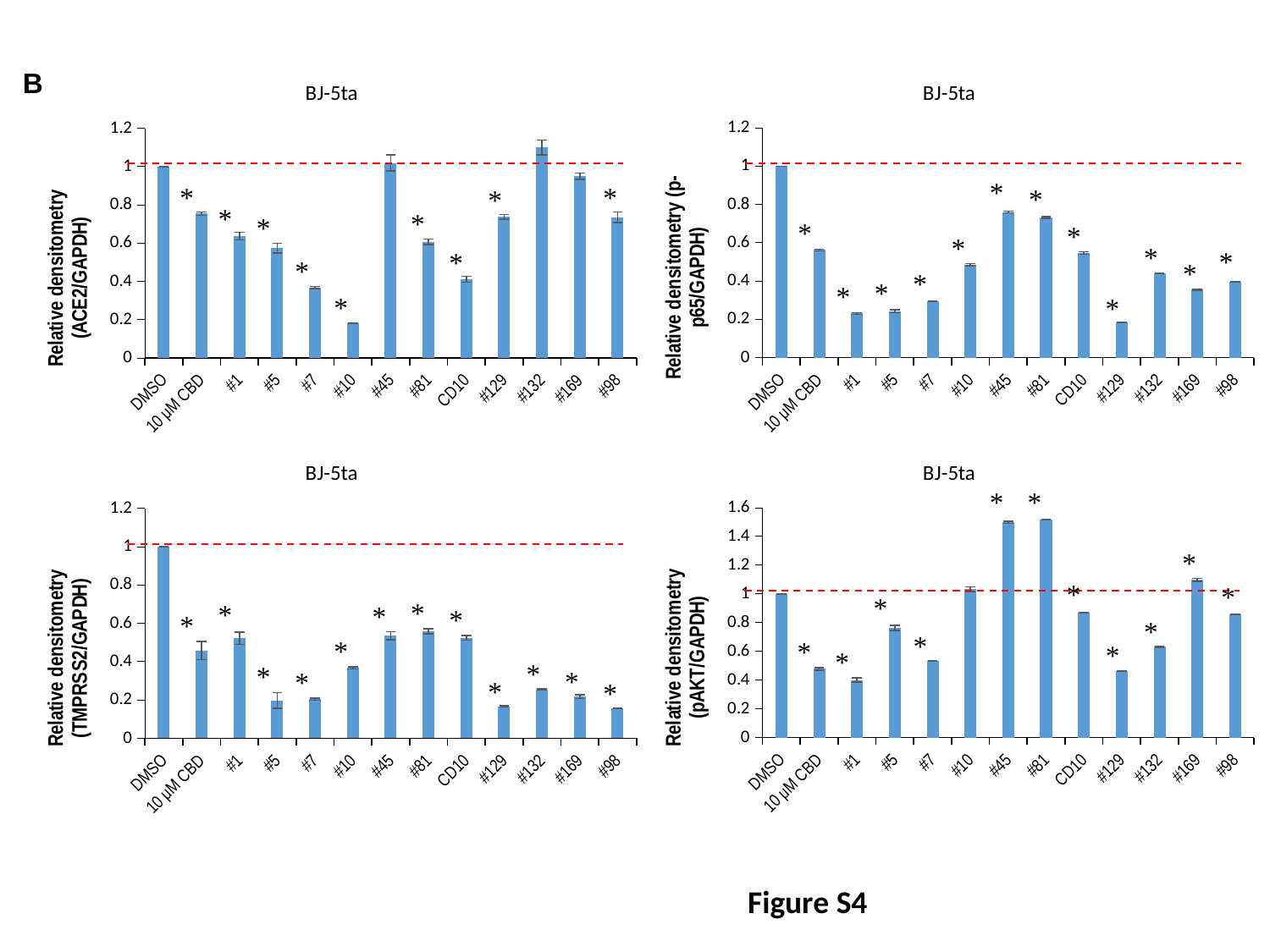

B
### Chart: BJ-5ta
| Category | |
|---|---|
| DMSO | 1.0 |
| 10 μM CBD | 0.5625310207502563 |
| #1 | 0.23086672199916283 |
| #5 | 0.24285161864283566 |
| #7 | 0.2955867900664908 |
| #10 | 0.4857257502376111 |
| #45 | 0.7608375199825497 |
| #81 | 0.7327353870573803 |
| CD10 | 0.5465306021616754 |
| #129 | 0.18223417237636327 |
| #132 | 0.44115809864029315 |
| #169 | 0.35506980486380024 |
| #98 | 0.3980141771731933 |*
*
*
*
*
*
*
*
*
*
*
*
### Chart: BJ-5ta
| Category | |
|---|---|
| DMSO | 1.0 |
| 10 μM CBD | 0.7540664814996916 |
| #1 | 0.6364468396079388 |
| #5 | 0.5743051942533547 |
| #7 | 0.36761286375279345 |
| #10 | 0.18233073445453032 |
| #45 | 1.0182016138428382 |
| #81 | 0.6068237482595871 |
| CD10 | 0.4103957412932846 |
| #129 | 0.7378329837056952 |
| #132 | 1.1000651778437214 |
| #169 | 0.9500314026677597 |
| #98 | 0.7356538457989055 |*
*
*
*
*
*
*
*
*
### Chart: BJ-5ta
| Category | |
|---|---|
| DMSO | 1.0 |
| 10 μM CBD | 0.4778252487535284 |
| #1 | 0.40002582748752125 |
| #5 | 0.7638652053486152 |
| #7 | 0.5325115283963557 |
| #10 | 1.032988985597283 |
| #45 | 1.499133924152369 |
| #81 | 1.5166482638797376 |
| CD10 | 0.8703715626193924 |
| #129 | 0.46401057034699333 |
| #132 | 0.6336278415975047 |
| #169 | 1.098794476620885 |
| #98 | 0.8571647464334892 |
### Chart: BJ-5ta
| Category | |
|---|---|
| DMSO | 1.0 |
| 10 μM CBD | 0.45810587728802 |
| #1 | 0.5222971906762301 |
| #5 | 0.1979191819065197 |
| #7 | 0.20467846826116626 |
| #10 | 0.36843601193691344 |
| #45 | 0.5356090131262965 |
| #81 | 0.5582627763051434 |
| CD10 | 0.5247770938989149 |
| #129 | 0.16721515891042885 |
| #132 | 0.25449257289379157 |
| #169 | 0.21740128746012344 |
| #98 | 0.15688956534179654 |*
*
*
*
*
*
*
*
*
*
*
*
*
*
*
*
*
*
*
*
*
*
*
Figure S4

## Slide 7
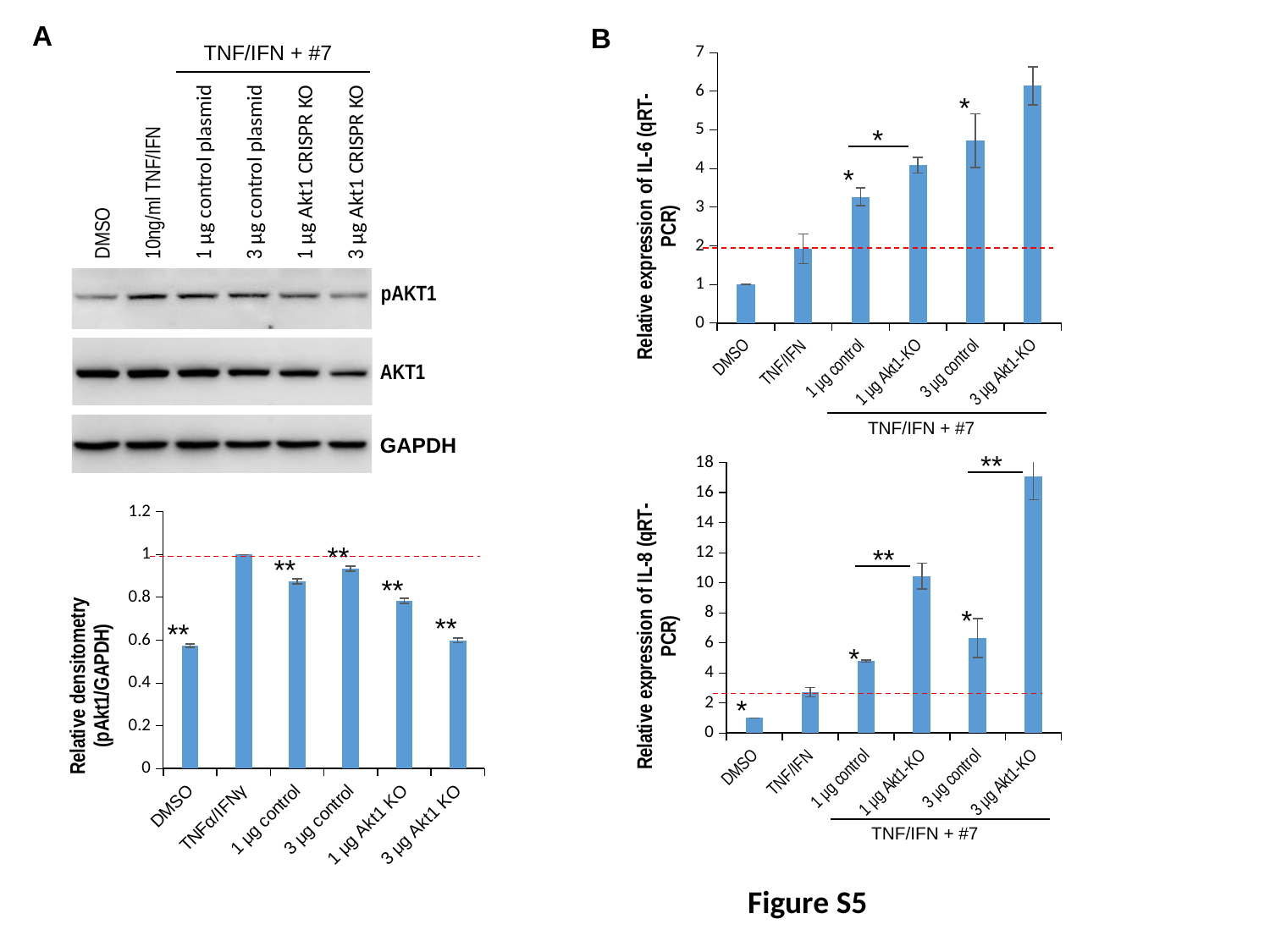

DMSO
10ng/ml TNF/IFN
1 μg control plasmid
3 μg control plasmid
1 μg Akt1 CRISPR KO
3 μg Akt1 CRISPR KO
A
B
TNF/IFN + #7
### Chart
| Category | |
|---|---|
| DMSO | 1.0 |
| TNF/IFN | 1.921878115215229 |
| 1 μg control | 3.2694469671960076 |
| 1 μg Akt1-KO | 4.082720558465493 |
| 3 μg control | 4.719043943356101 |
| 3 μg Akt1-KO | 6.138884329139182 |*
*
*
pAKT1
AKT1
TNF/IFN + #7
GAPDH
**
### Chart
| Category | |
|---|---|
| DMSO | 1.0 |
| TNF/IFN | 2.722195301439921 |
| 1 μg control | 4.779192881103056 |
| 1 μg Akt1-KO | 10.44601740220304 |
| 3 μg control | 6.3100013256697425 |
| 3 μg Akt1-KO | 17.05747985947072 |
### Chart
| Category | |
|---|---|
| DMSO | 0.5736748467016687 |
| TNFα/IFNγ | 1.0 |
| 1 μg control | 0.8750296640087024 |
| 3 μg control | 0.9344897080867738 |
| 1 μg Akt1 KO | 0.7840254079350681 |
| 3 μg Akt1 KO | 0.5988931921845637 |**
**
**
**
*
**
**
*
*
TNF/IFN + #7
Figure S5

## Slide 8
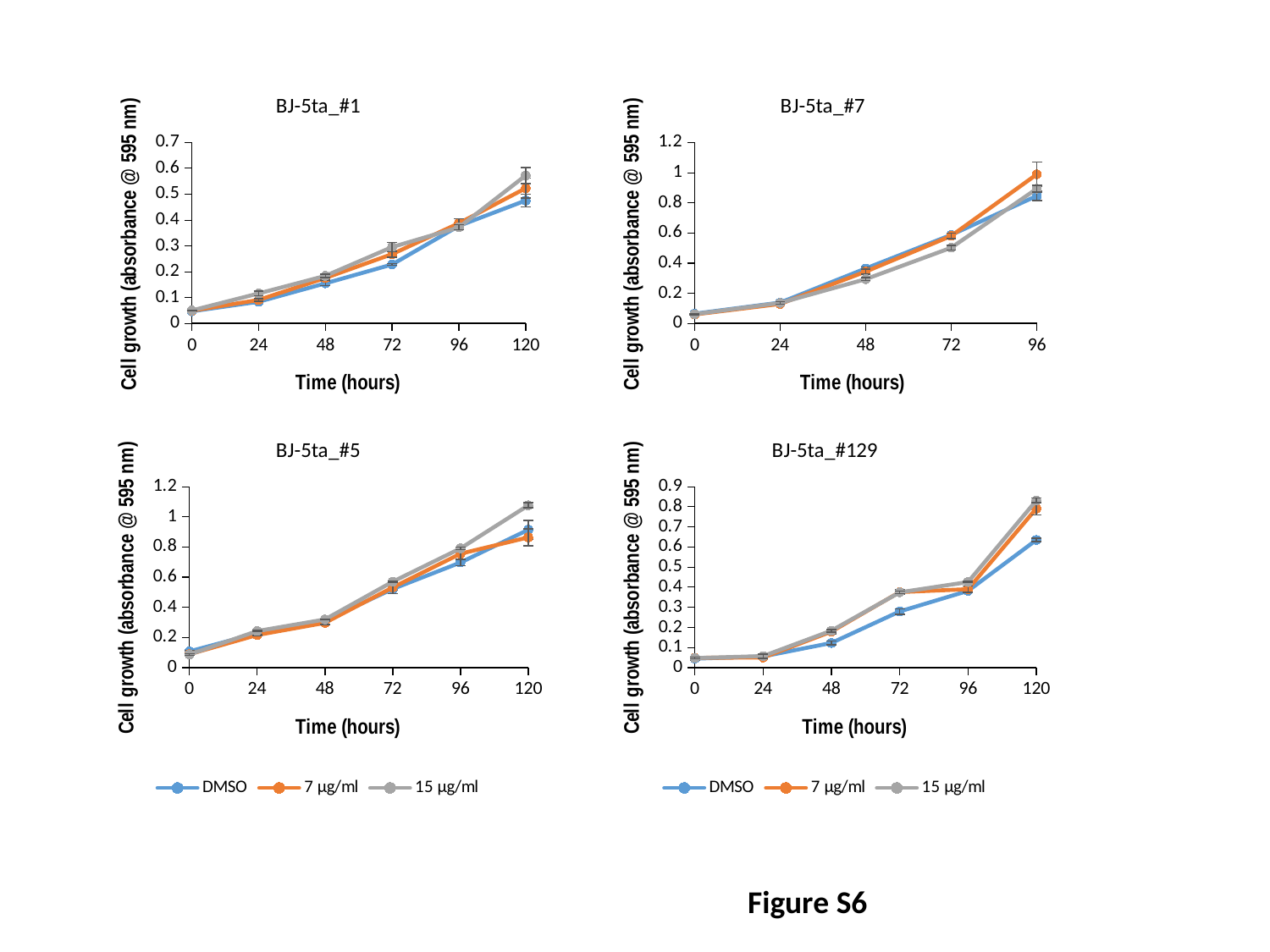

### Chart: BJ-5ta_#1
| Category | DMSO | 7 μg/ml | 15 μg/ml |
|---|---|---|---|
| 0 | 0.04699999999999999 | 0.0494333333333333 | 0.050333333333333334 |
| 24 | 0.084 | 0.091 | 0.11599999999999999 |
| 48 | 0.1551666666666667 | 0.176 | 0.18400000000000005 |
| 72 | 0.22833333333333336 | 0.268366666666667 | 0.2956666666666667 |
| 96 | 0.3775 | 0.387777777777777 | 0.3718333333333333 |
| 120 | 0.4756666666666667 | 0.523333333333333 | 0.5726666666666667 |
### Chart: BJ-5ta_#7
| Category | DMSO | 7 µg/ml | 15 µg/ml |
|---|---|---|---|
| 0 | 0.06466666666666668 | 0.05933333333333335 | 0.061666666666666675 |
| 24 | 0.13833333333333334 | 0.12933333333333336 | 0.13599999999999998 |
| 48 | 0.36466666666666664 | 0.3436666666666666 | 0.29366666666666663 |
| 72 | 0.5876666666666667 | 0.5806666666666668 | 0.5023333333333334 |
| 96 | 0.846 | 0.989 | 0.8938333333333333 |
### Chart: BJ-5ta_#5
| Category | DMSO | 7 μg/ml | 15 μg/ml |
|---|---|---|---|
| 0 | 0.10766666666666665 | 0.08899999999999998 | 0.08666666666666667 |
| 24 | 0.22766666666666668 | 0.21633333333333335 | 0.24233333333333332 |
| 48 | 0.3116666666666666 | 0.2968333333333333 | 0.3193333333333333 |
| 72 | 0.5216666666666666 | 0.532 | 0.57 |
| 96 | 0.6973333333333334 | 0.7556666666666666 | 0.7908333333333332 |
| 120 | 0.9141666666666666 | 0.864 | 1.0761666666666667 |
### Chart: BJ-5ta_#129
| Category | DMSO | 7 μg/ml | 15 μg/ml |
|---|---|---|---|
| 0 | 0.04466666666666666 | 0.048666666666666664 | 0.04766666666666666 |
| 24 | 0.05566666666666667 | 0.050333333333333334 | 0.057333333333333326 |
| 48 | 0.12266666666666666 | 0.17900000000000002 | 0.18366666666666664 |
| 72 | 0.2793333333333333 | 0.3758333333333333 | 0.37466666666666665 |
| 96 | 0.3818333333333333 | 0.39033333333333325 | 0.4258333333333333 |
| 120 | 0.6349999999999999 | 0.7915 | 0.8313333333333333 |Figure S6

## Slide 9
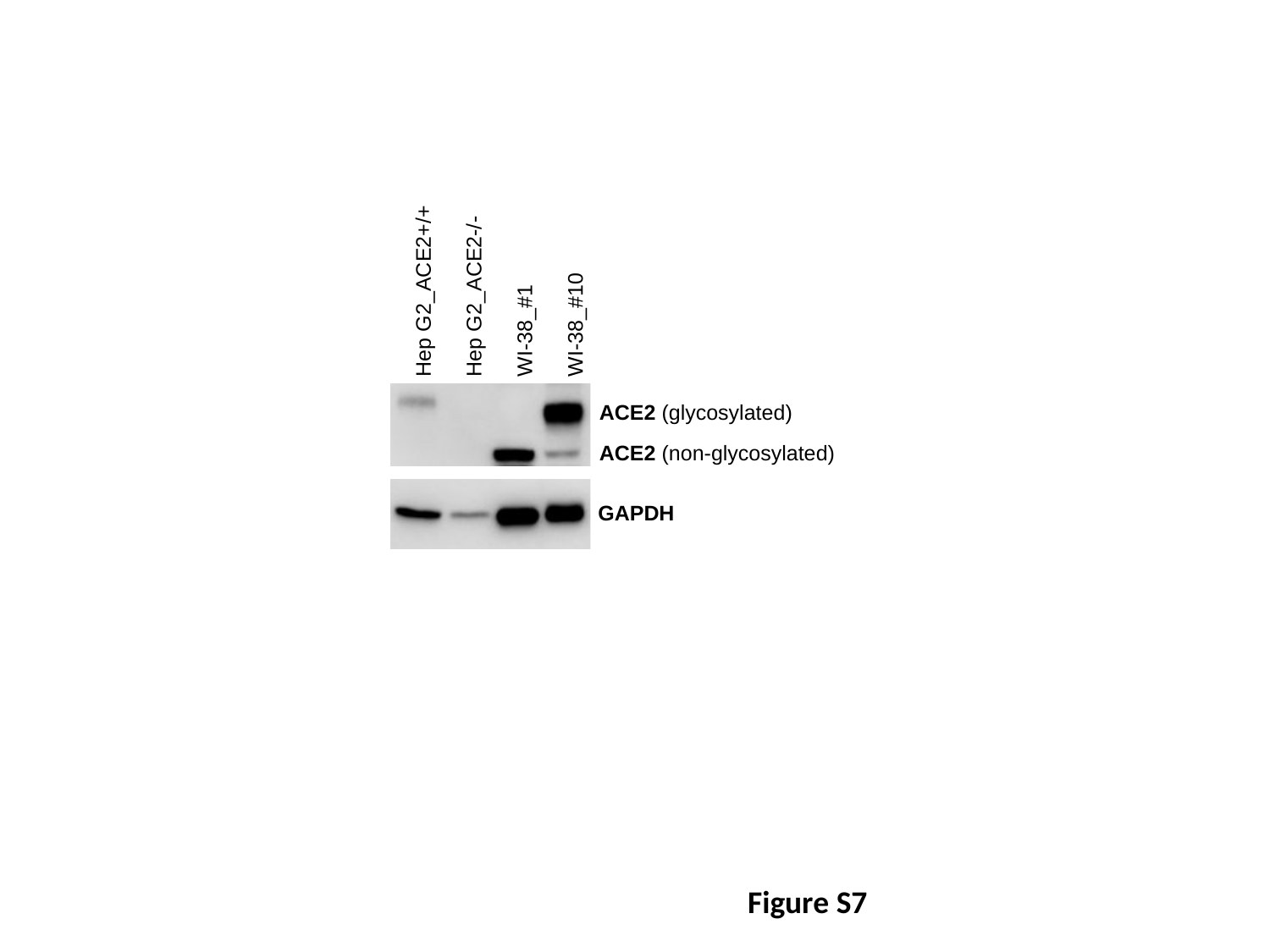

Hep G2_ACE2+/+
Hep G2_ACE2-/-
WI-38_#1
WI-38_#10
ACE2 (glycosylated)
ACE2 (non-glycosylated)
GAPDH
Figure S7
