# Supplementary material for: New AKT-dependent mechanisms of anti-COVID-19 action of high-CBD Cannabis sativa extracts
Source: Cell Death Discov. 2022 Mar 11;8:110. doi: 10.1038/s41420-022-00876-y (PMC8913855; doi:10.1038/s41420-022-00876-y)

Original image: Figure 1A

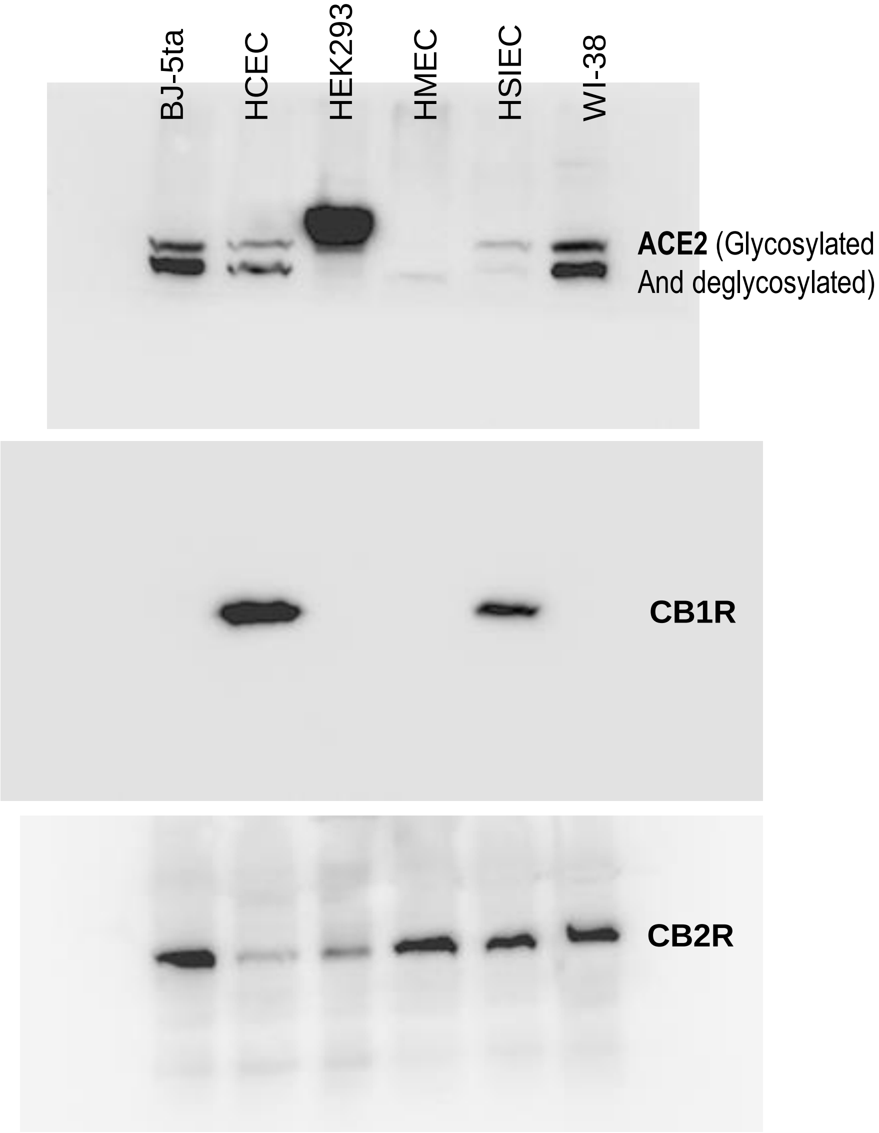

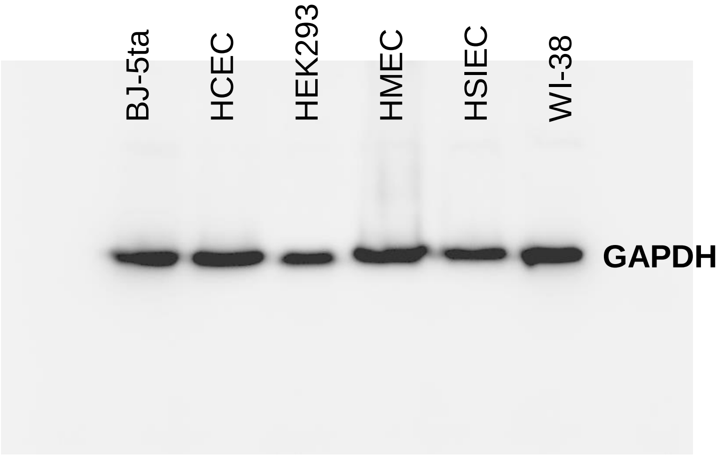

Original image: Figure 1B and 1C

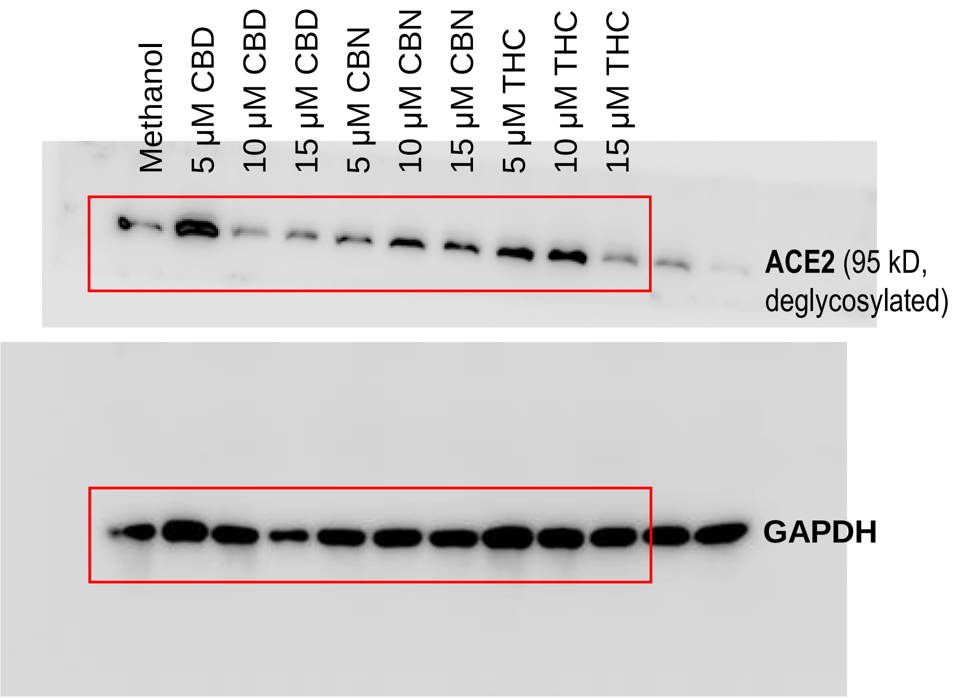

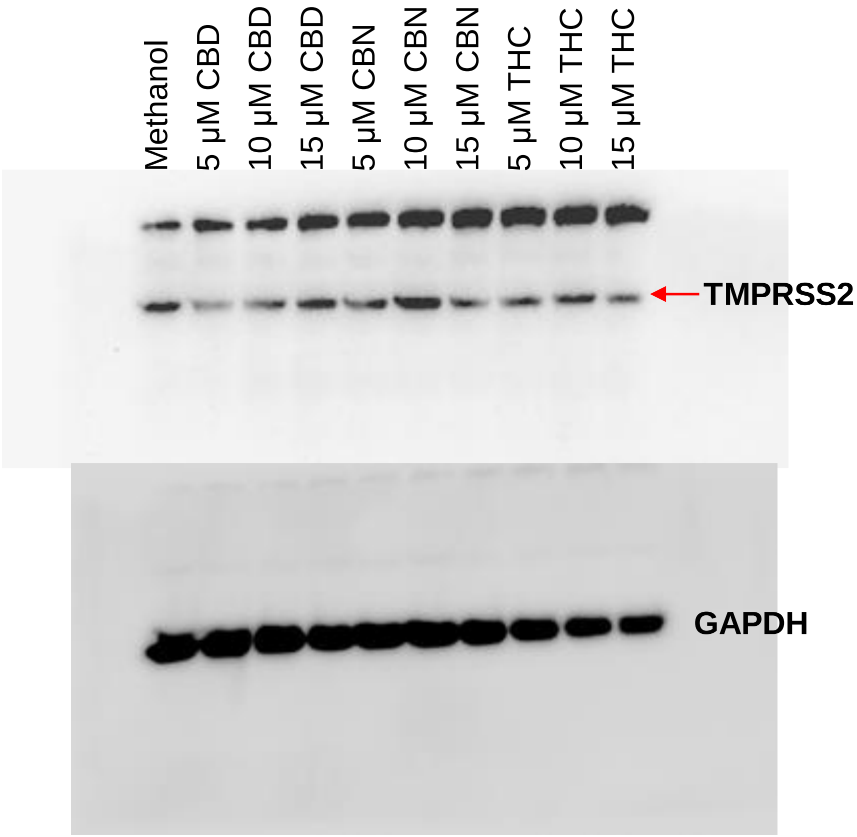

Original image: Figure 1D

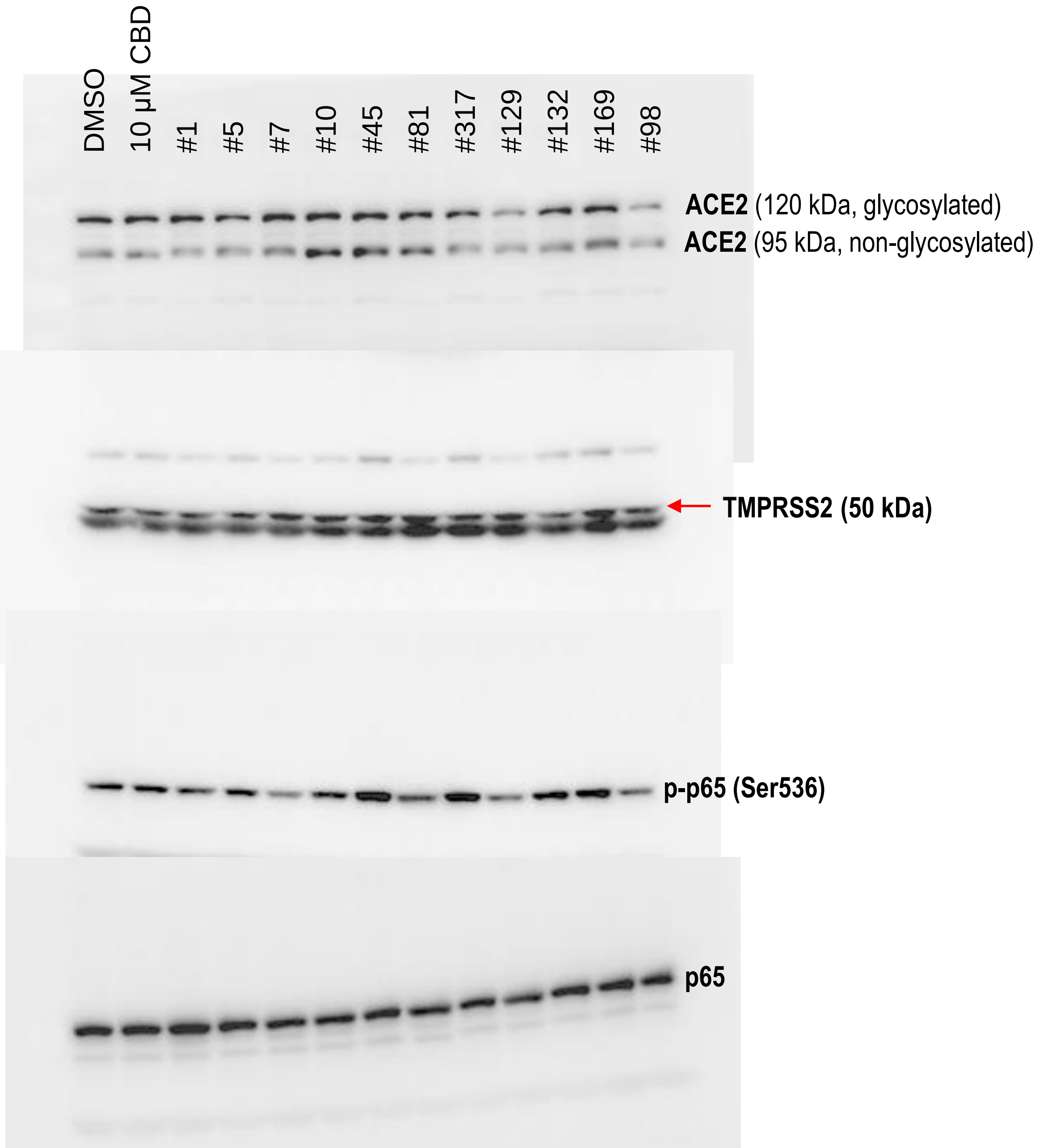

Original image: Figure 1D

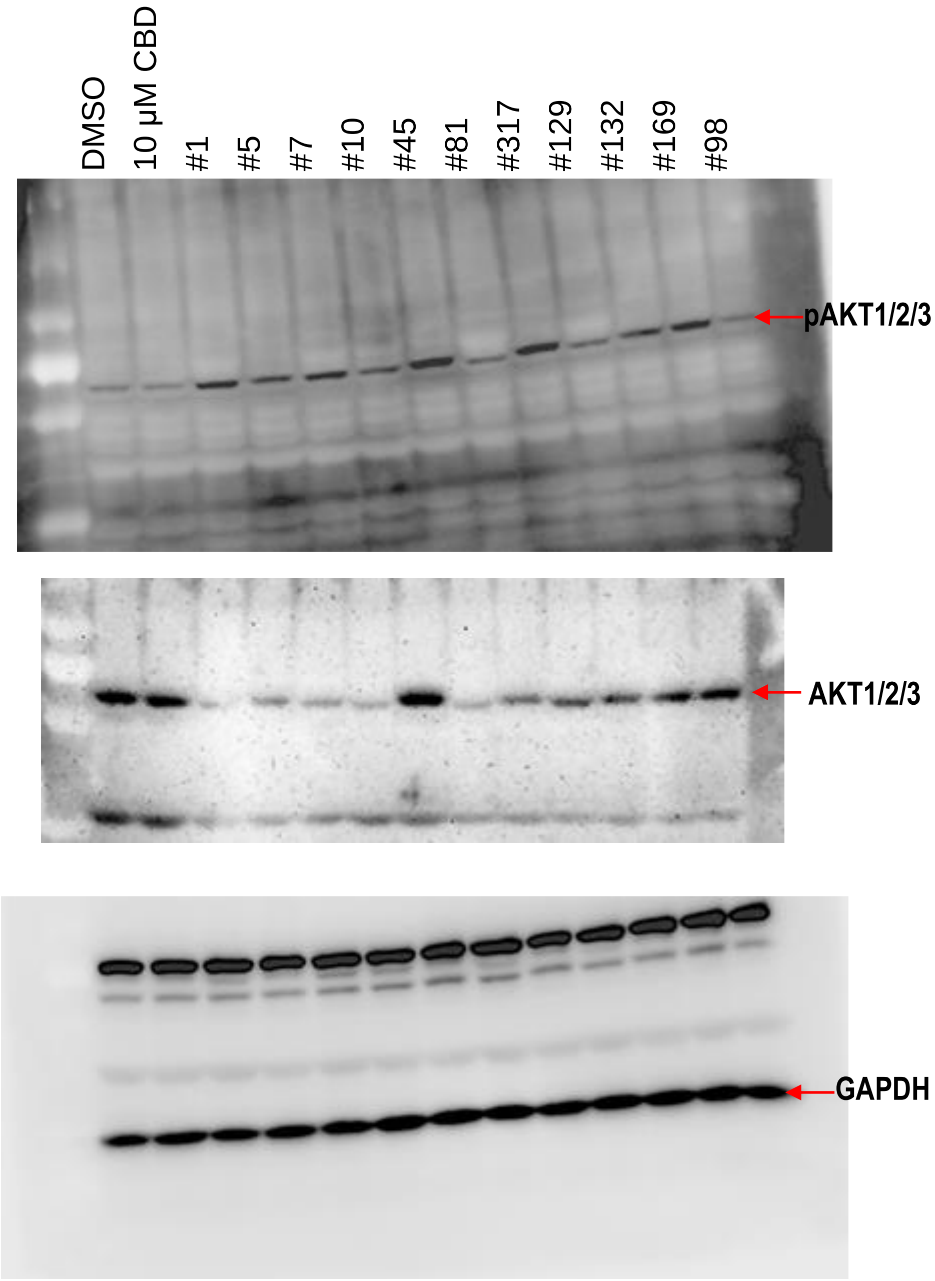

Original image: Figure 1E

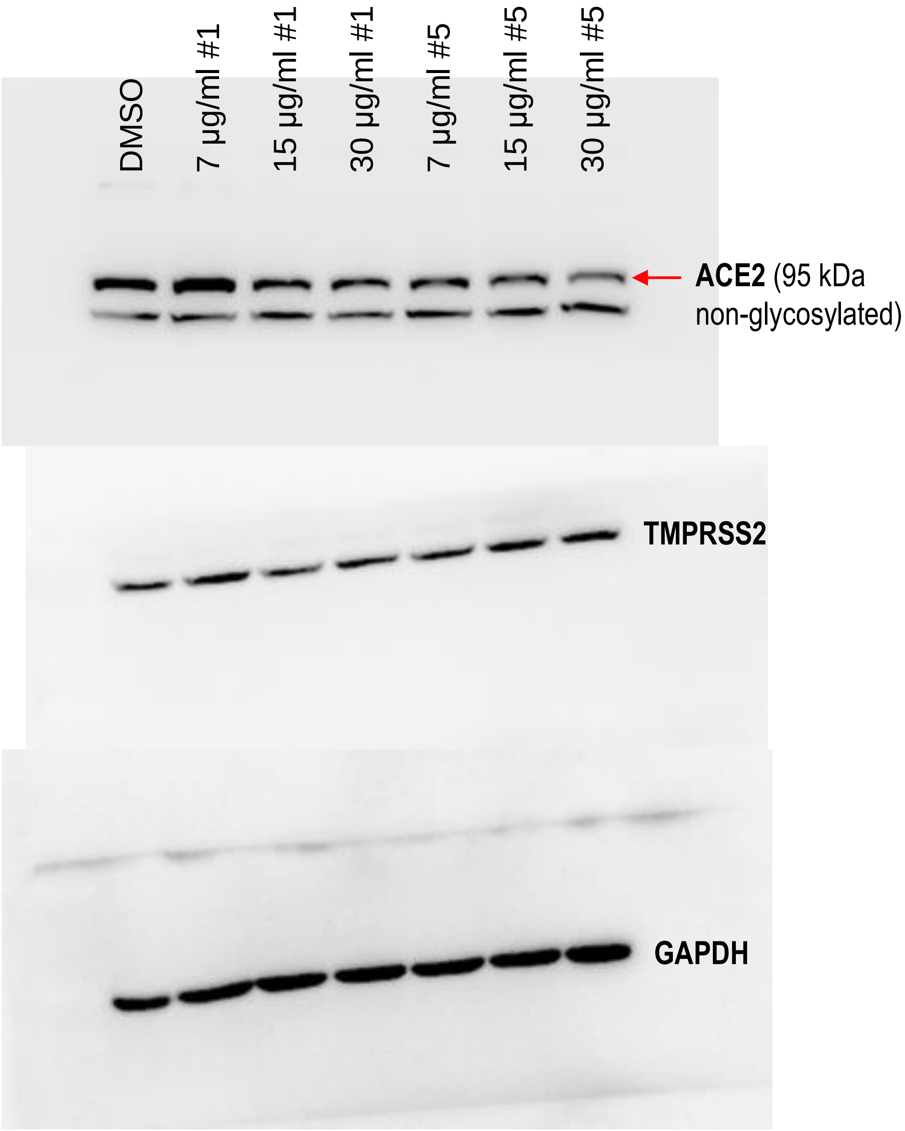

Original image: Figure 2E

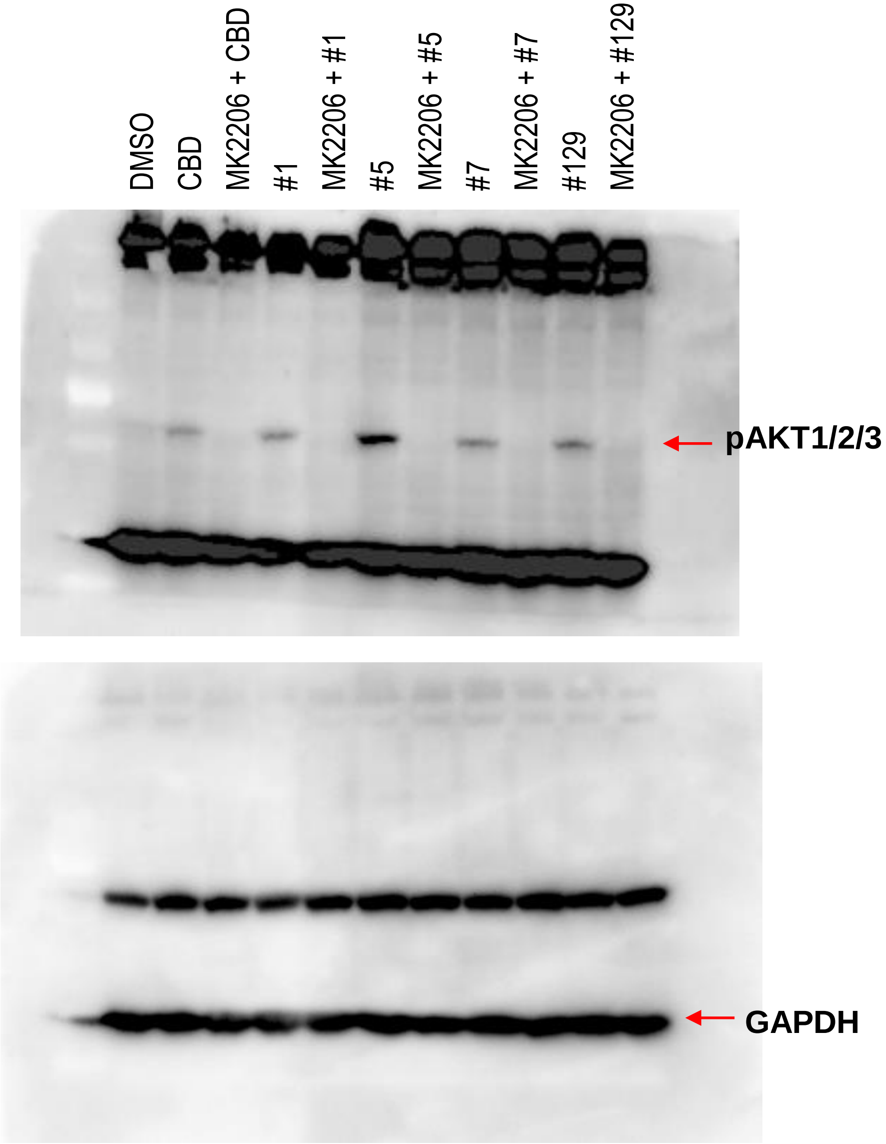

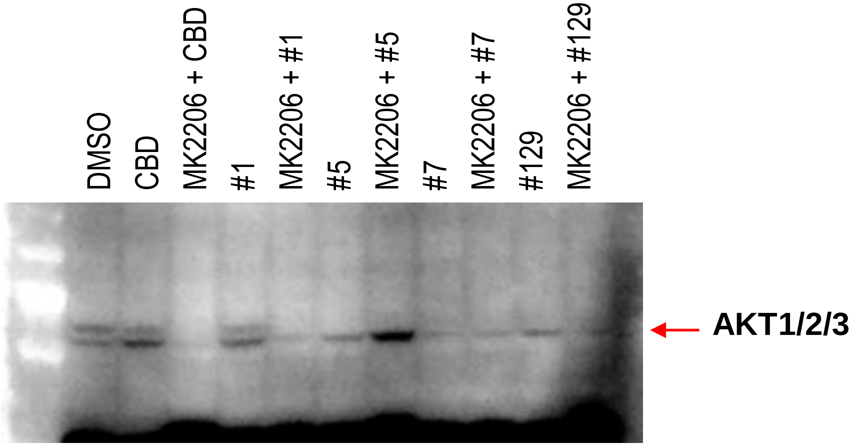

Original image: Figure 3A

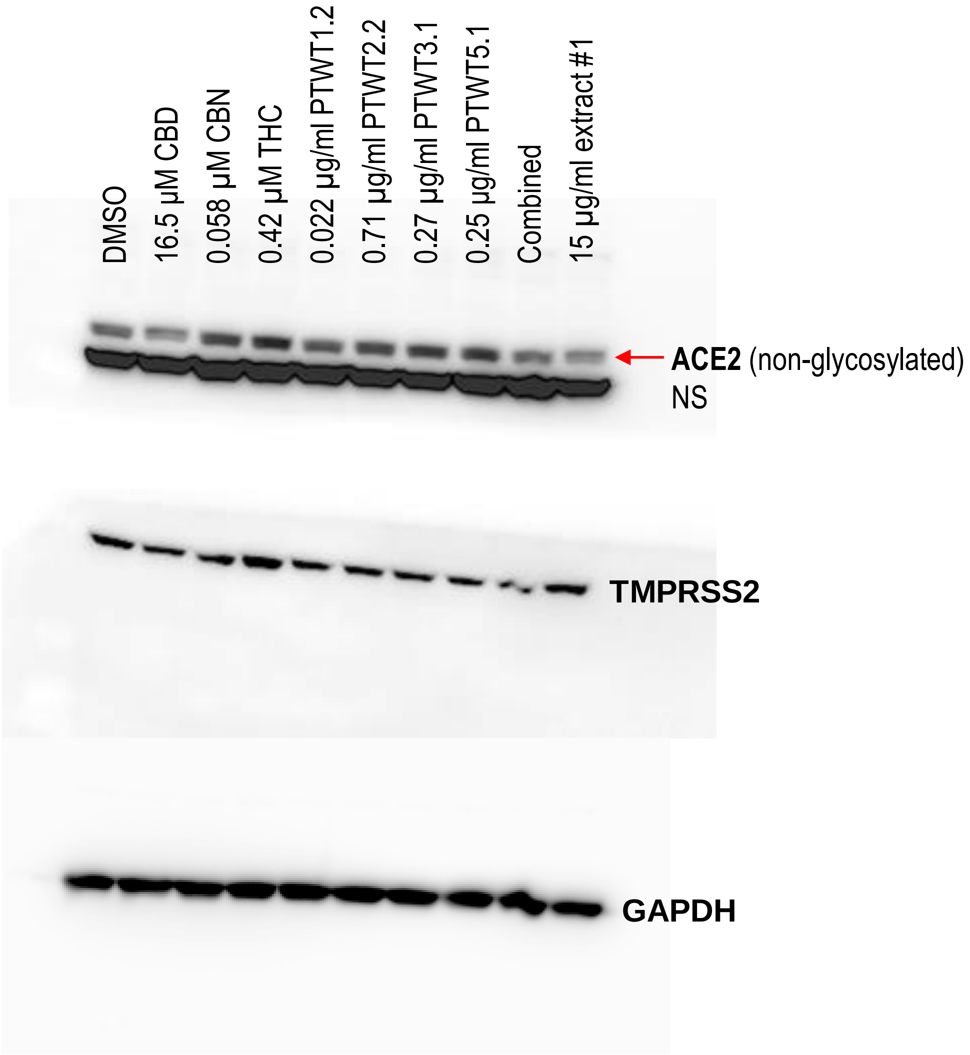

Original image: Figure 3B

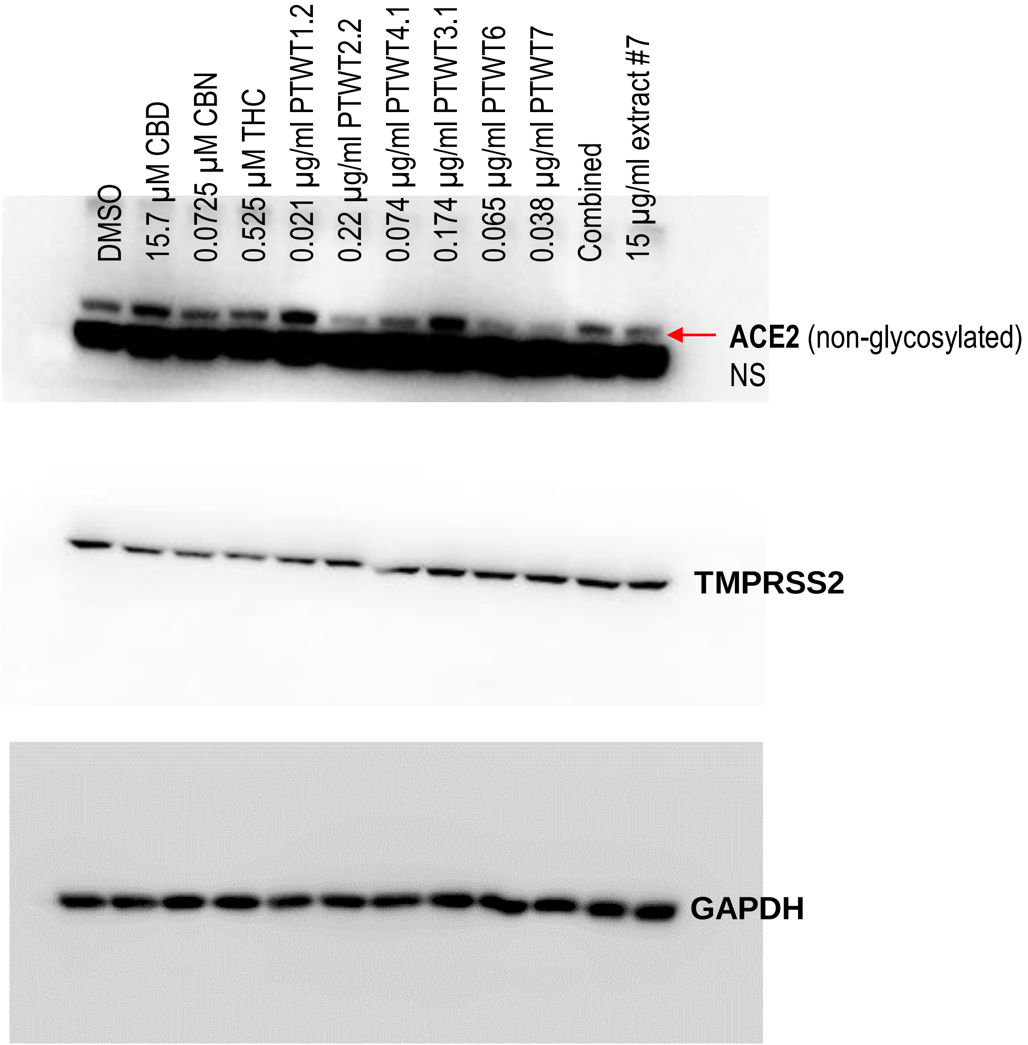

Original image: Figure 3C

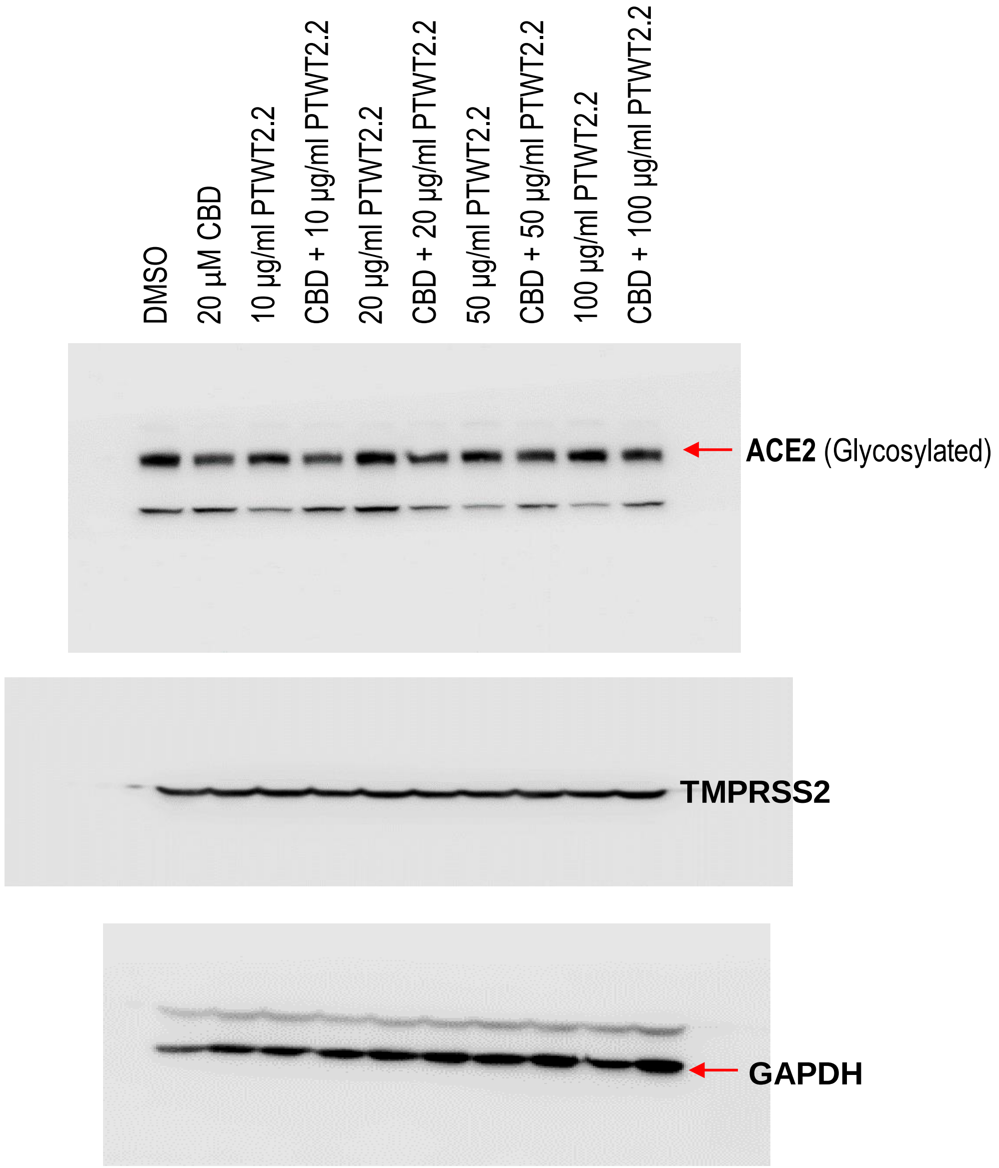

Original image: Figure 3D

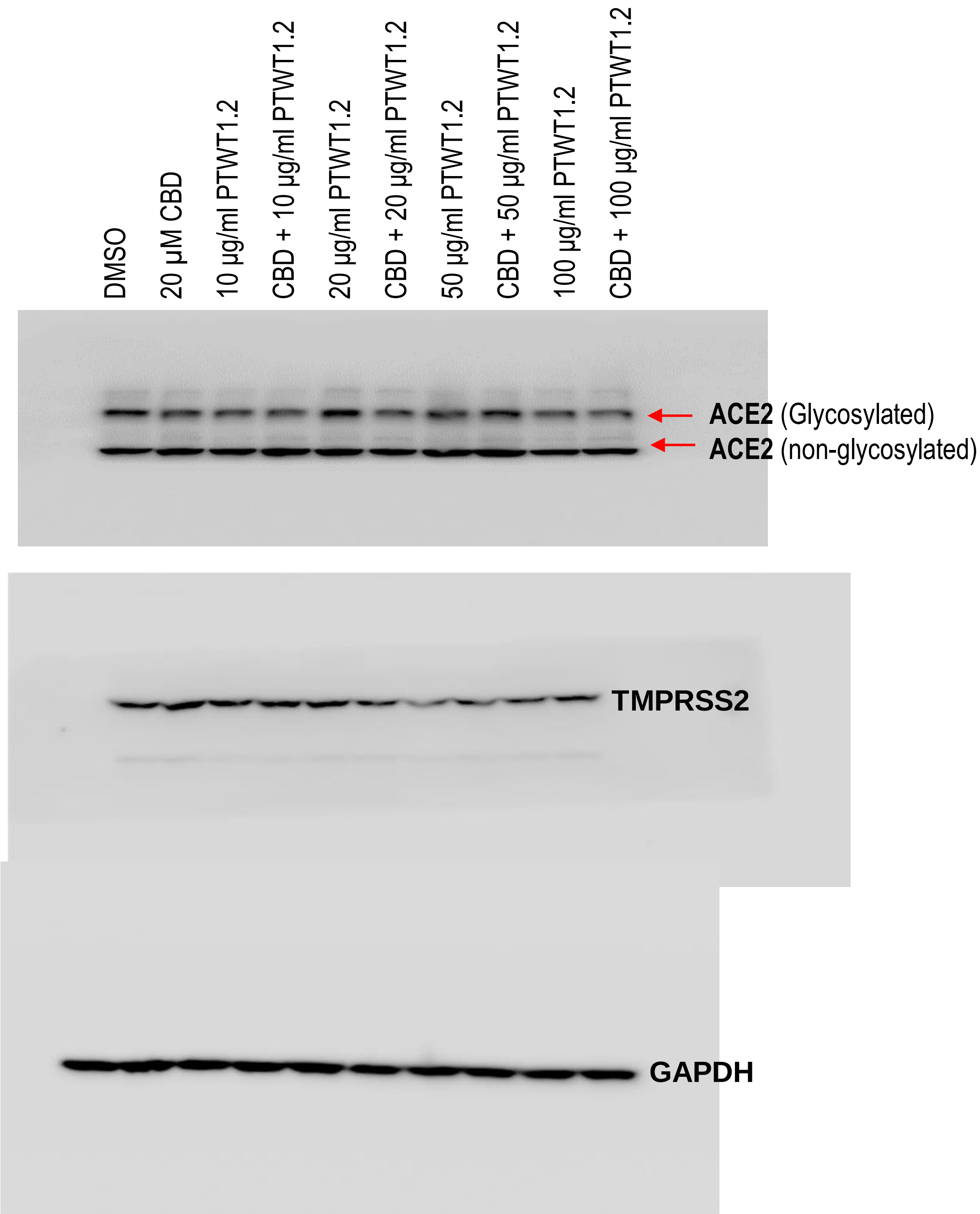

Original image: Figure 4A

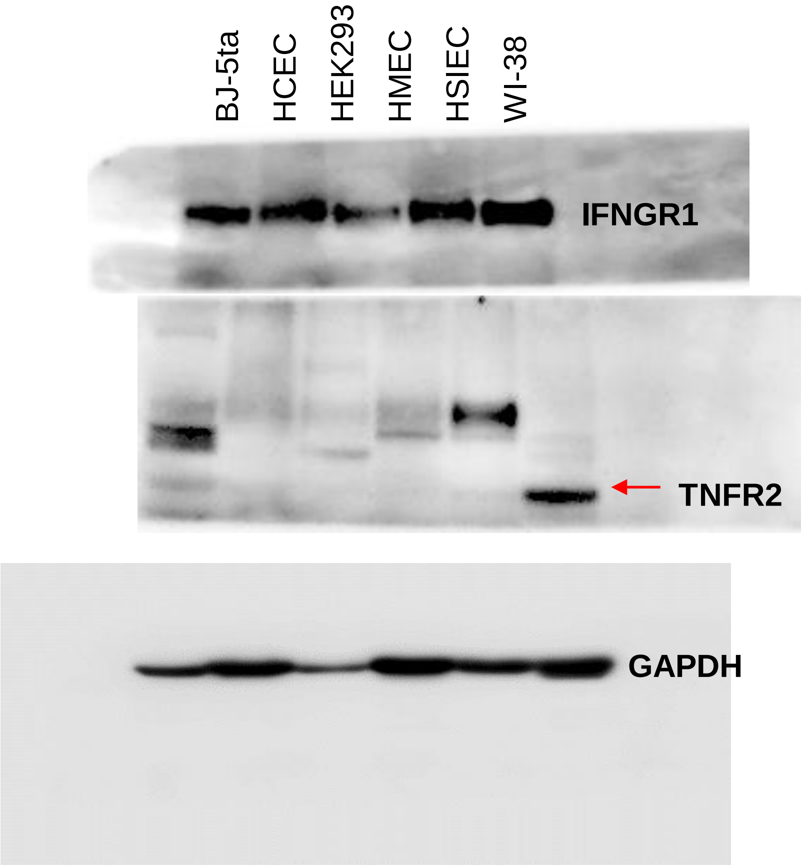

Original image: Figure 4C and 4D

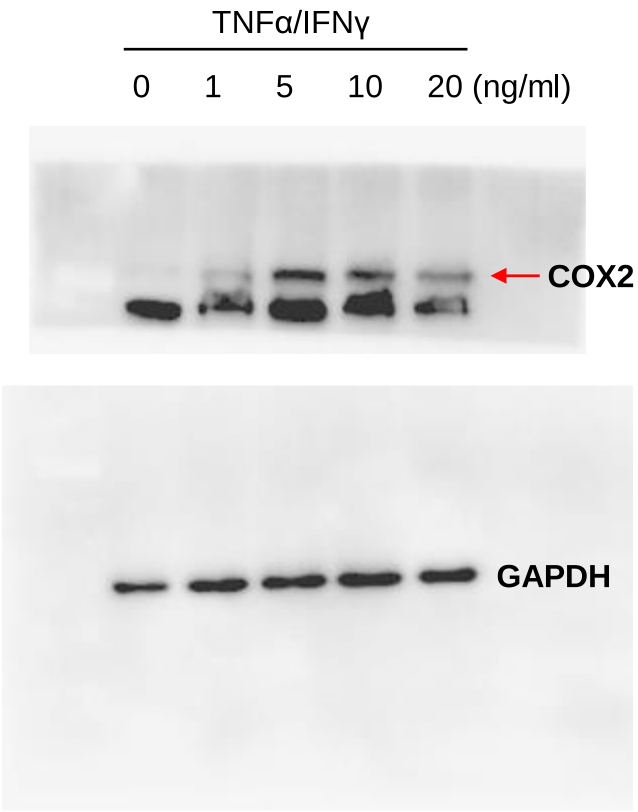

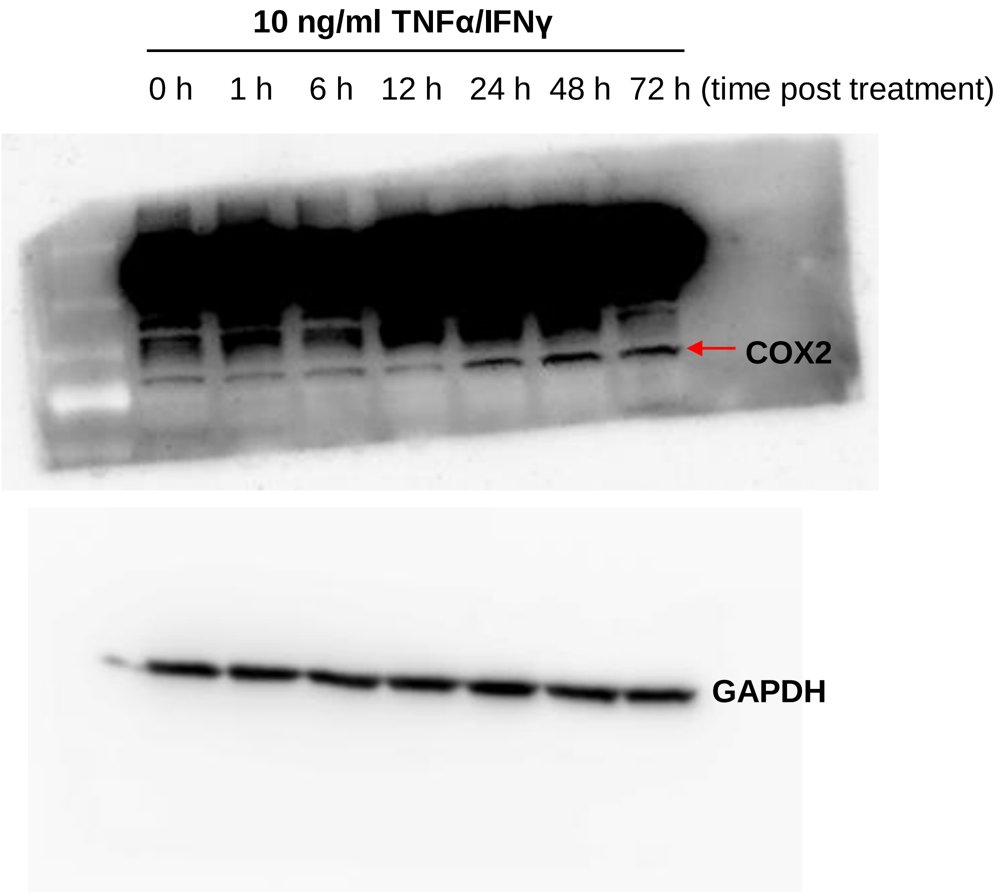

Original image: Figure 4E and 4F

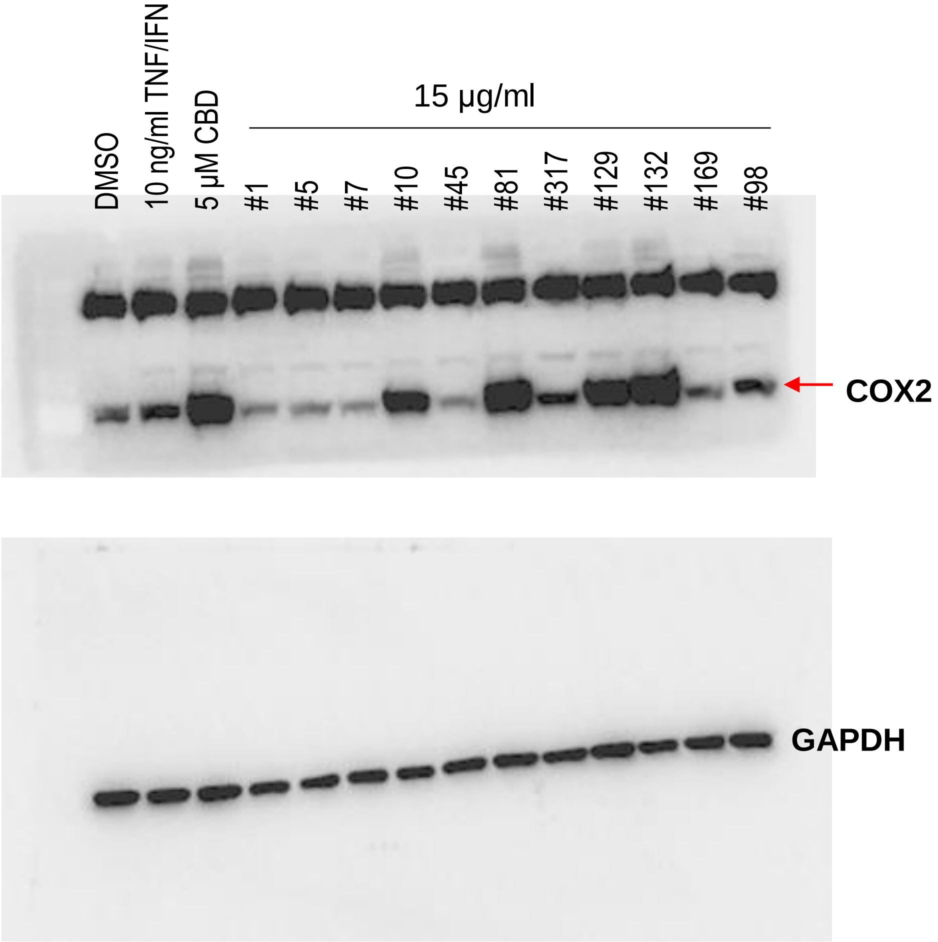

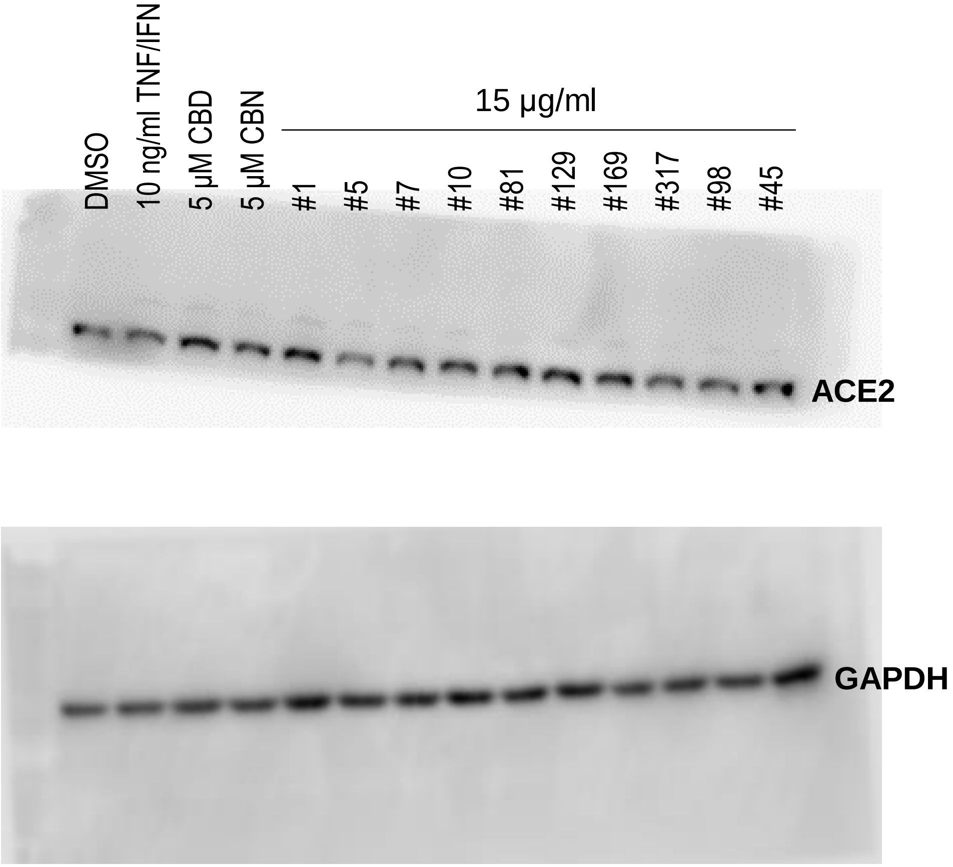

Original image: Figure 5A

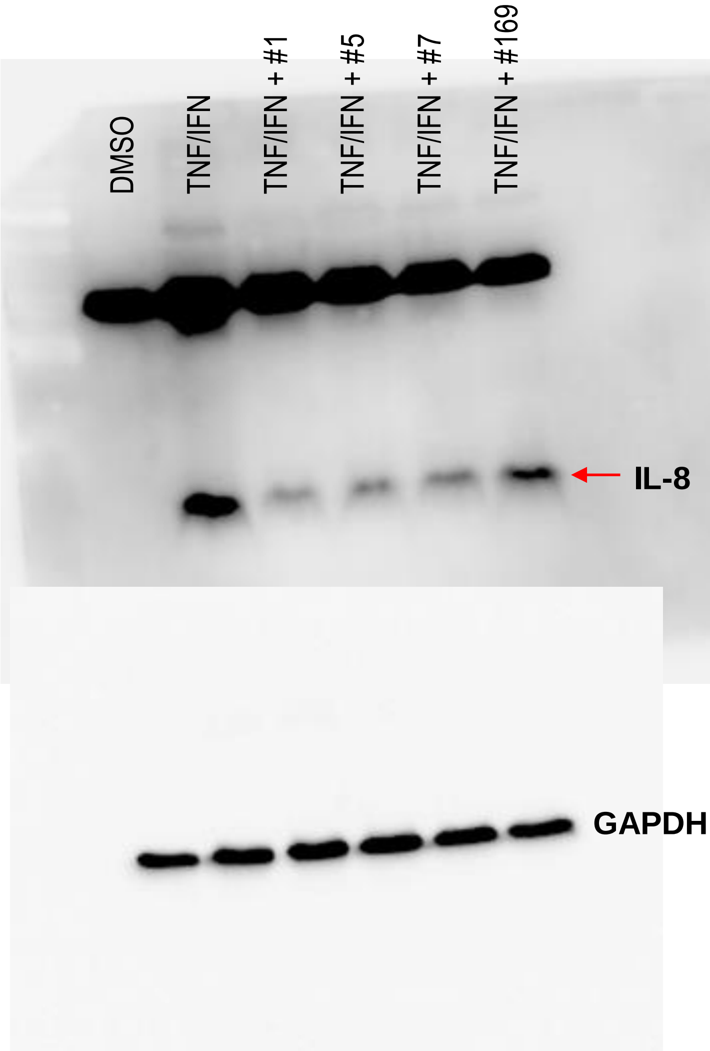

Original image: Figure 5B

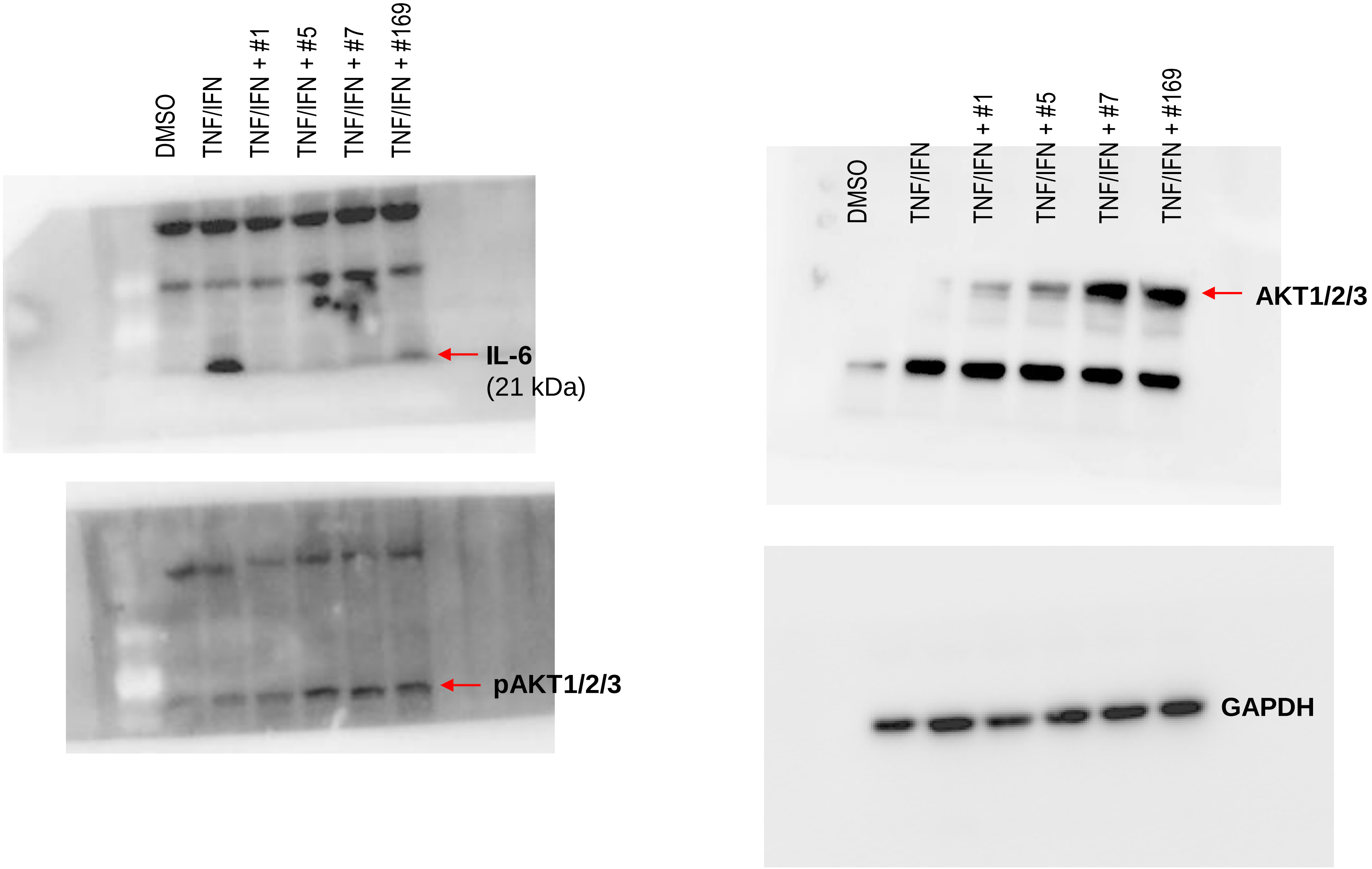

Original image: Figure 5C

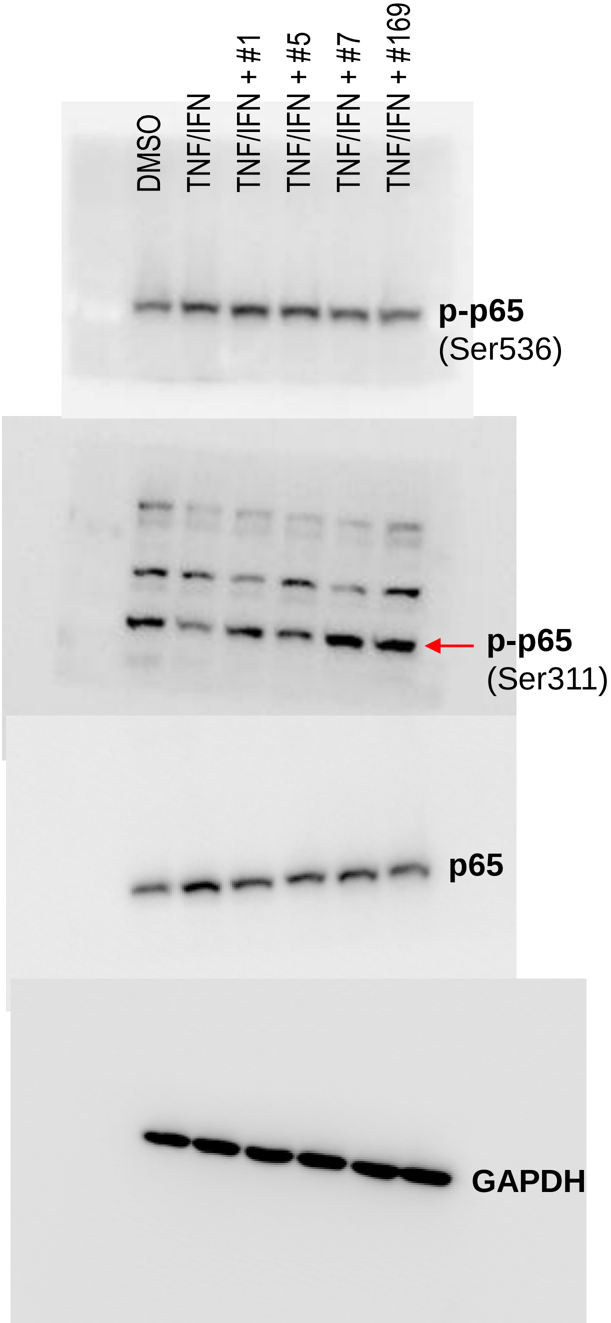

Original image: Figure 5G

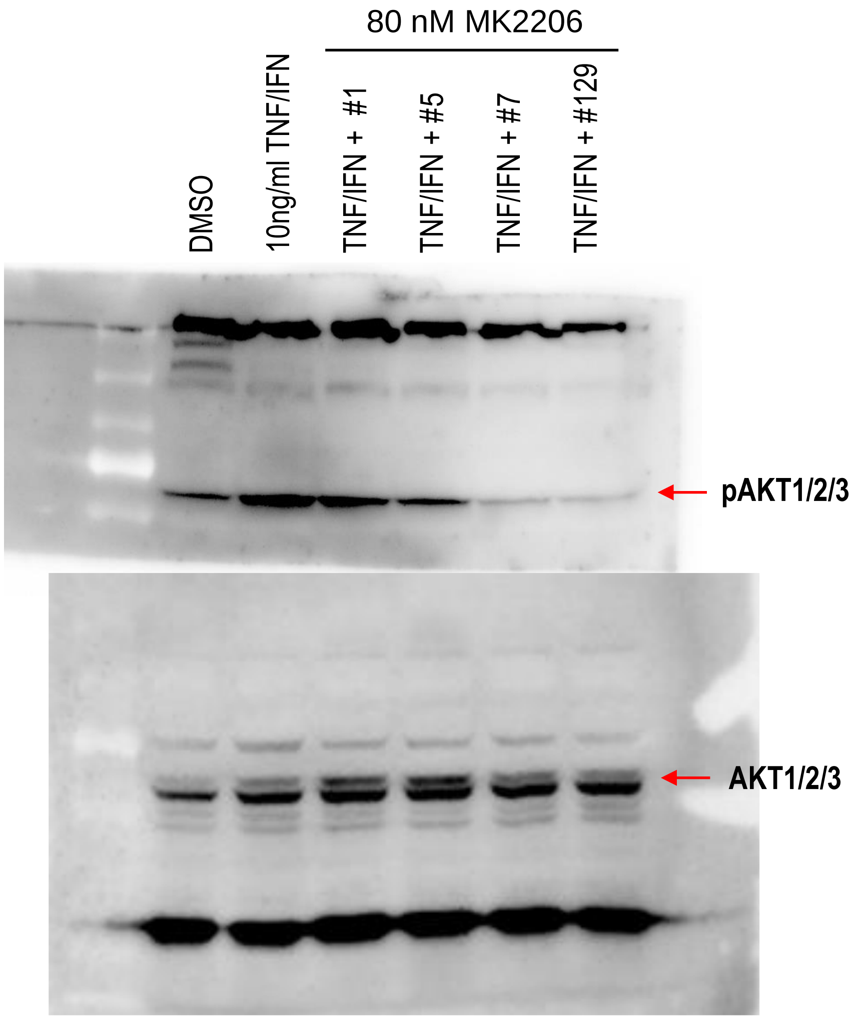

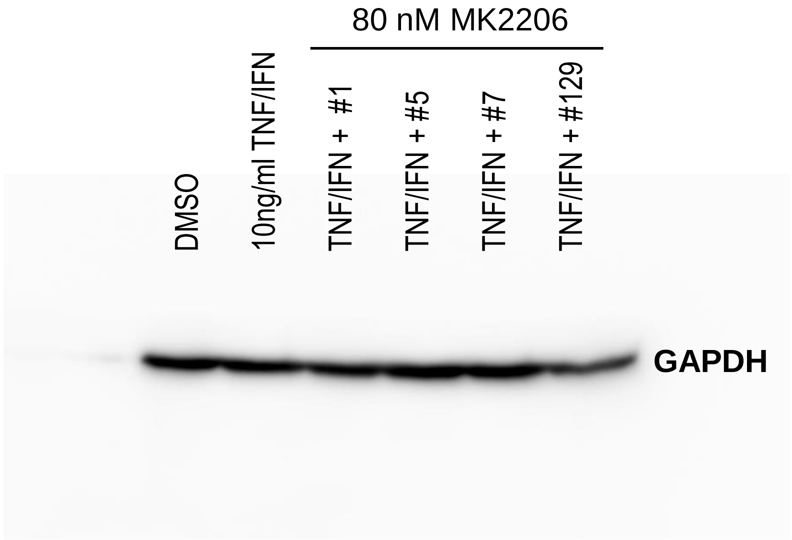

Original image: Figure 6A

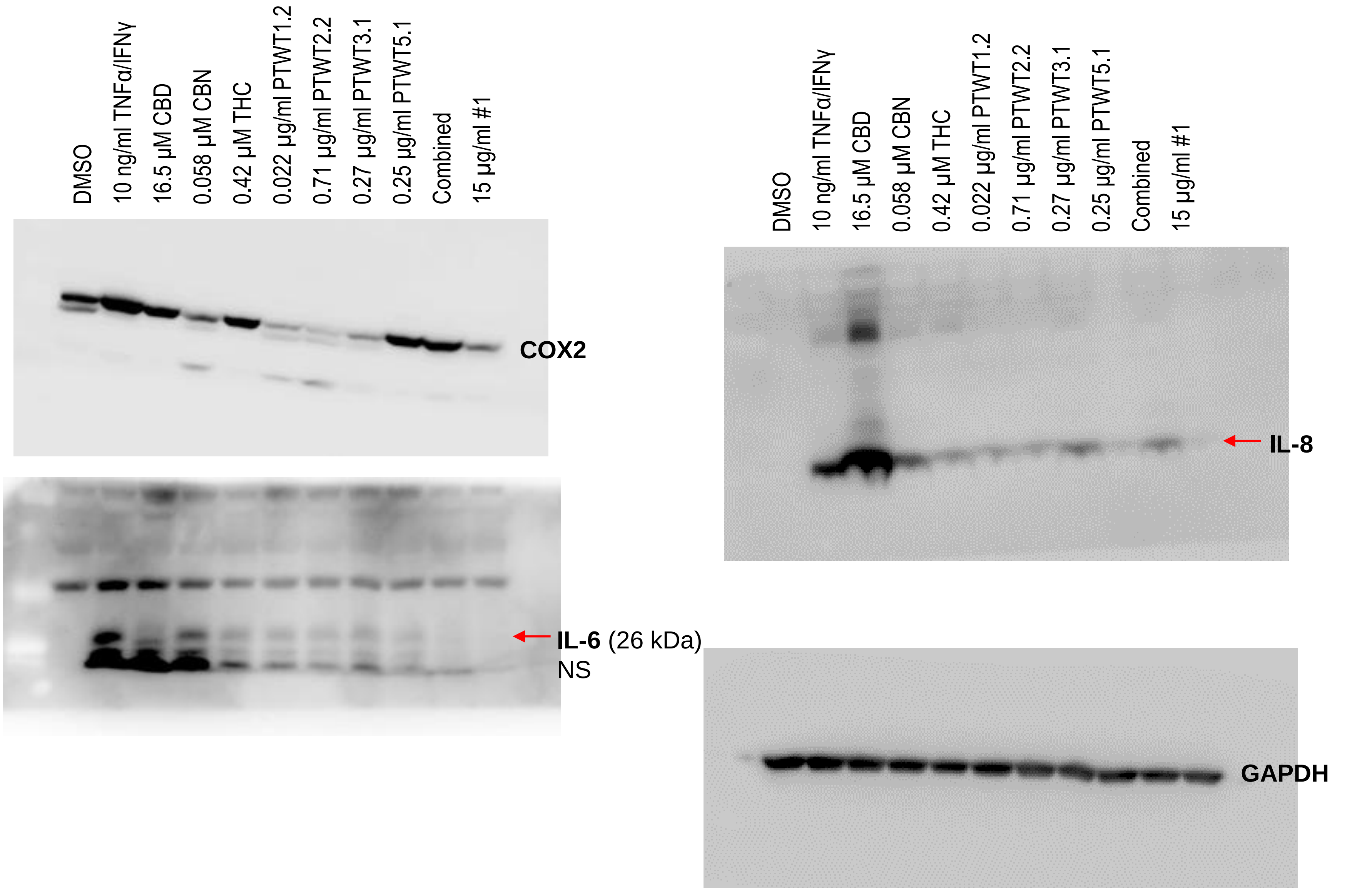

Original image: Figure 6B

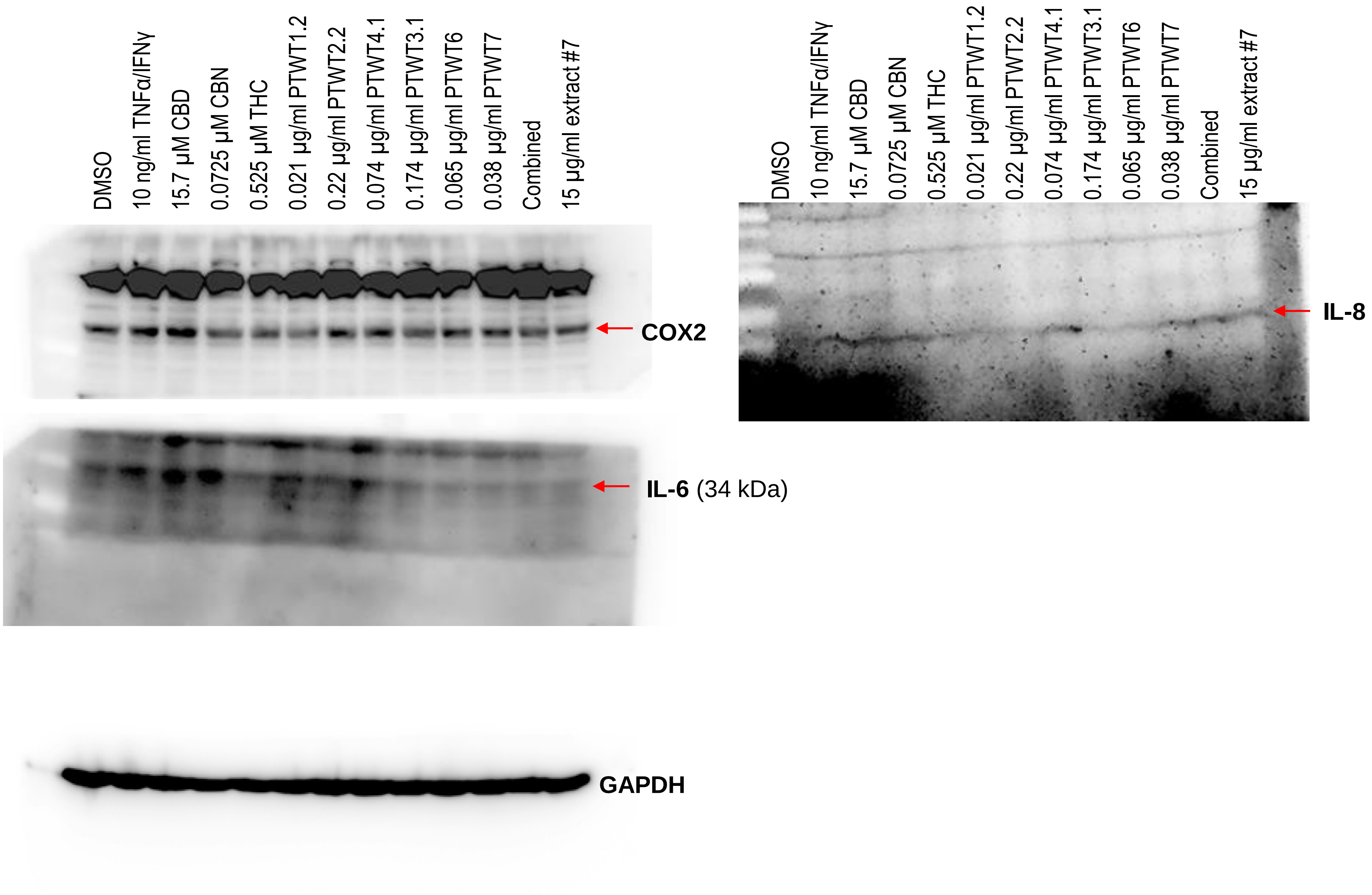

Original image: Figure S3A and S3B

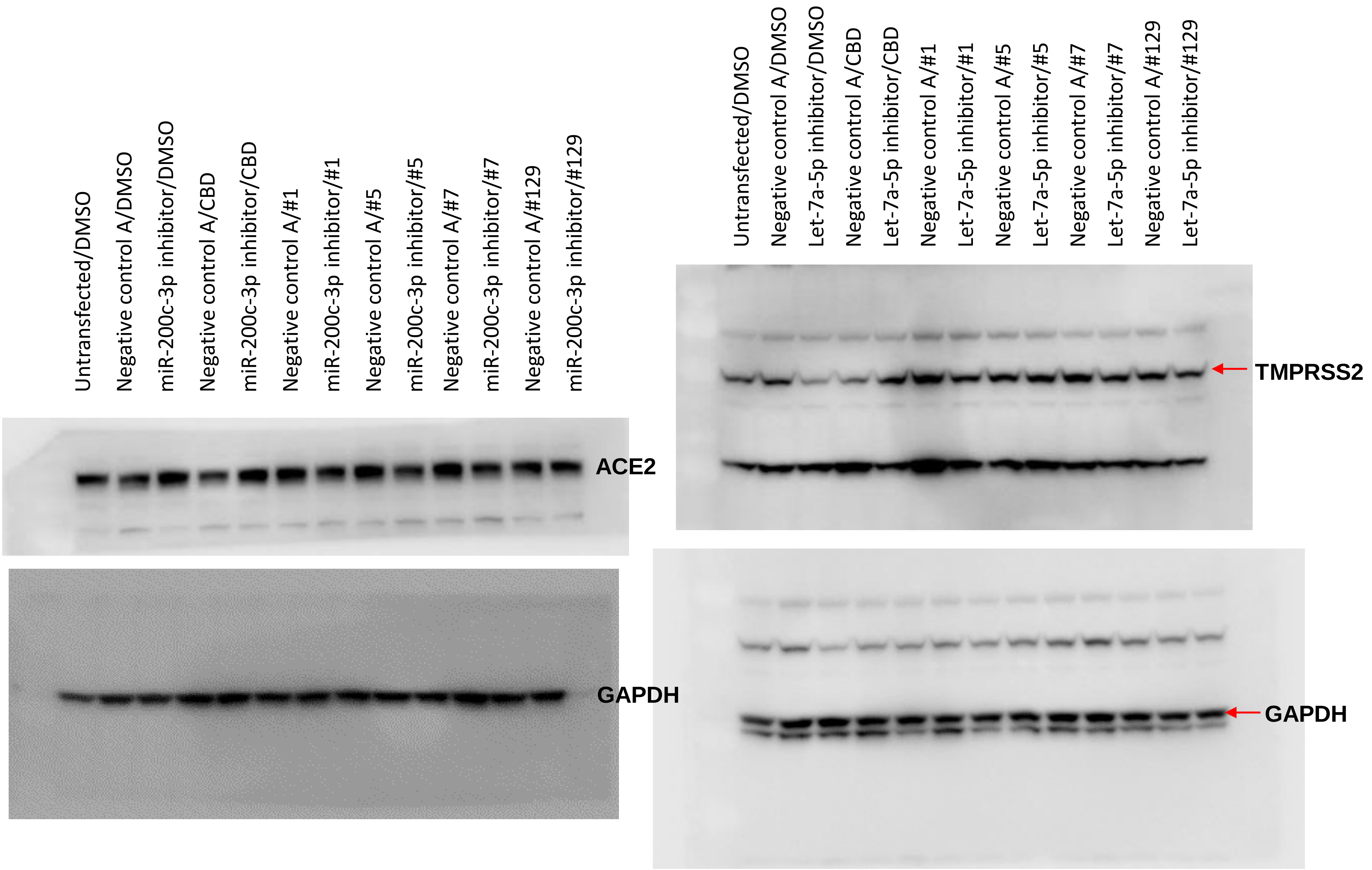

Original image: Figure S4A

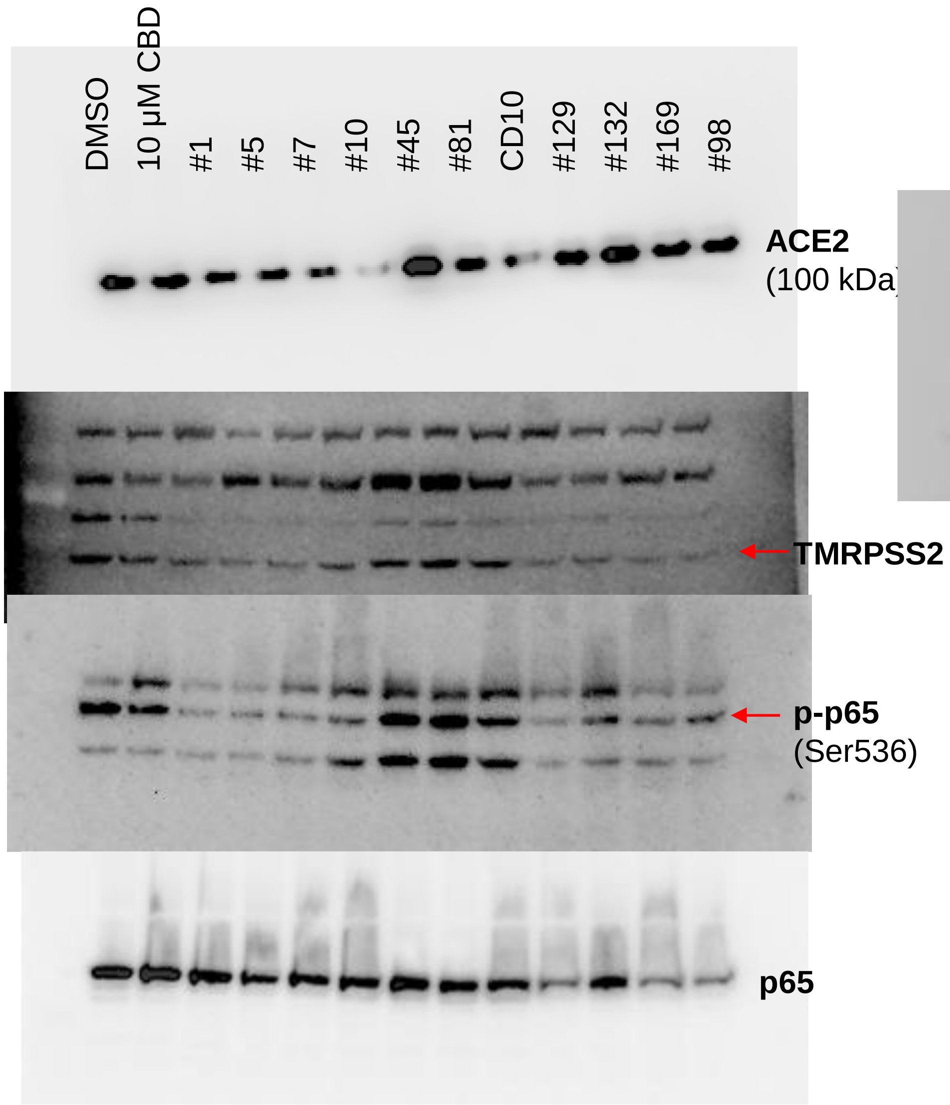

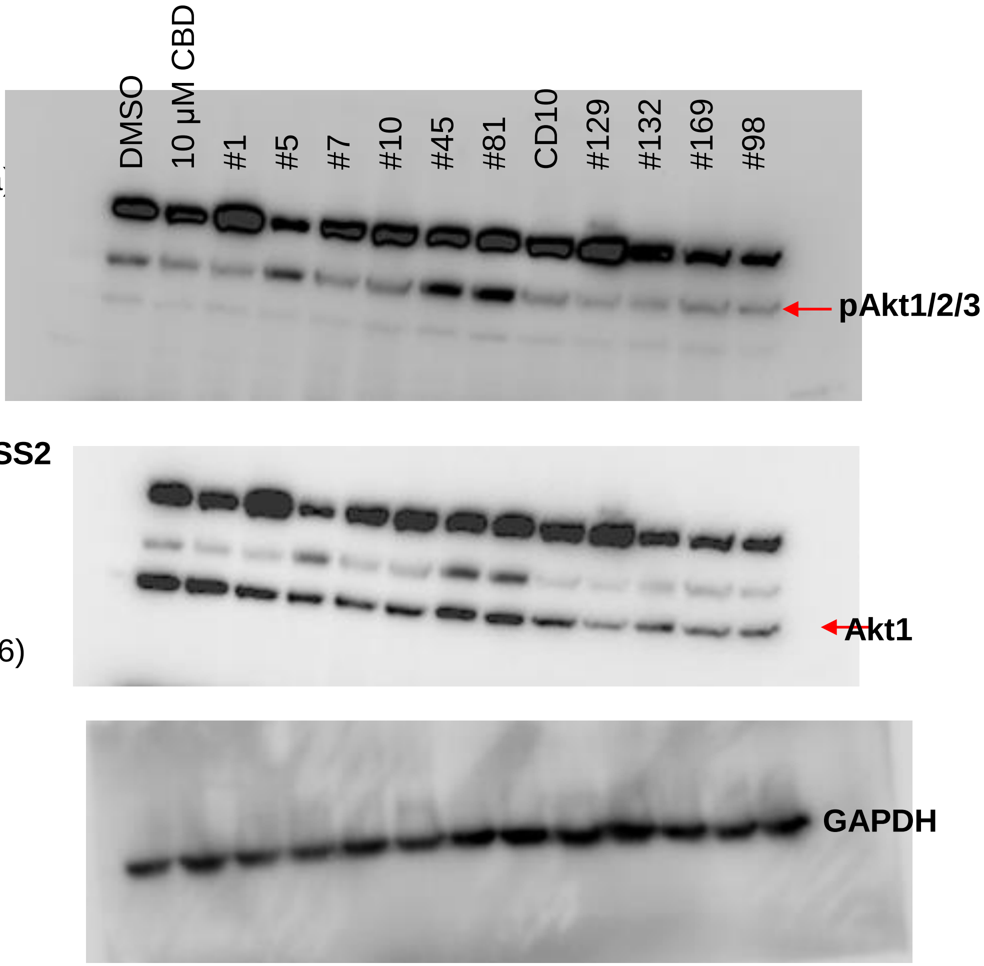

Original image: Figure S5A

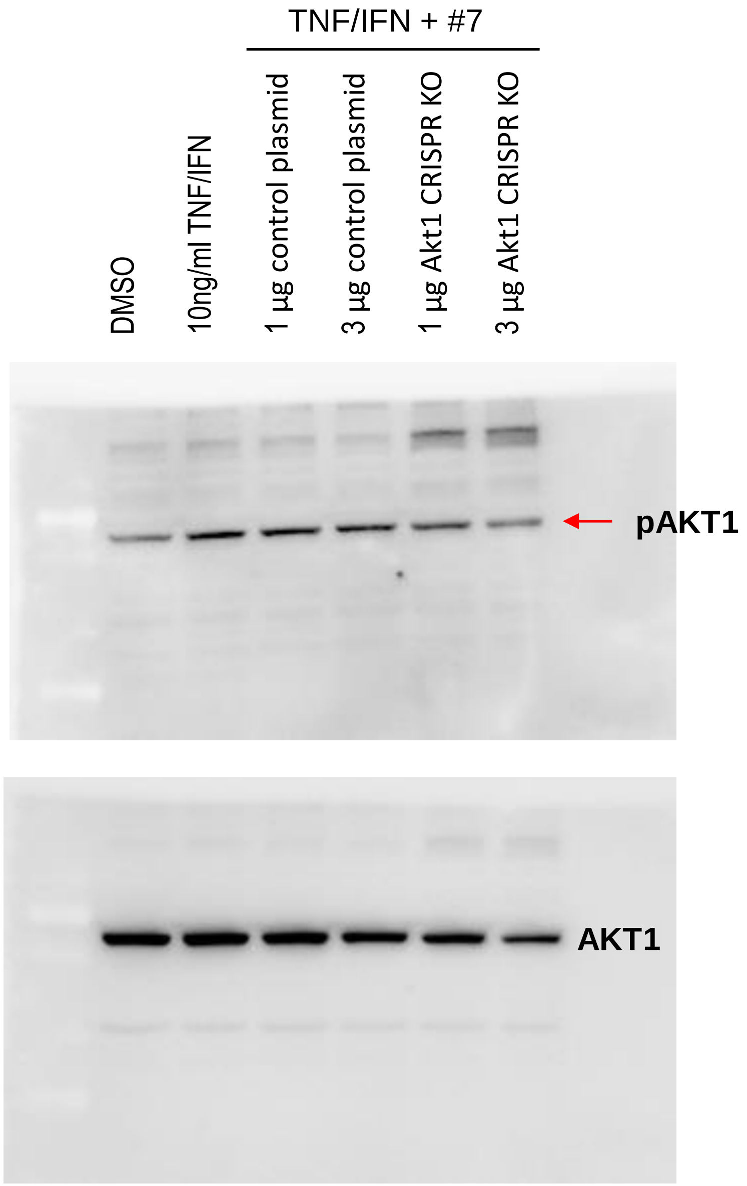

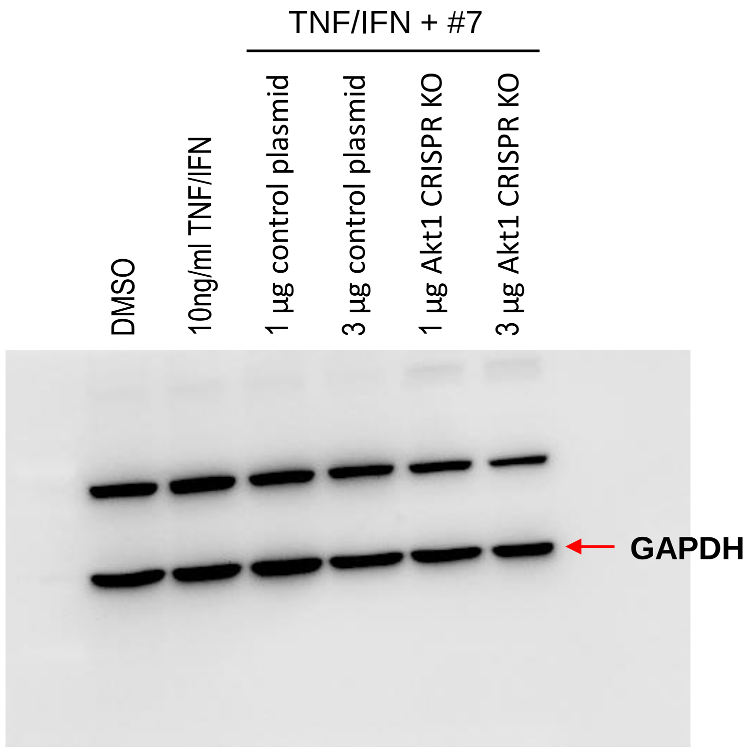

Original image: Figure S7

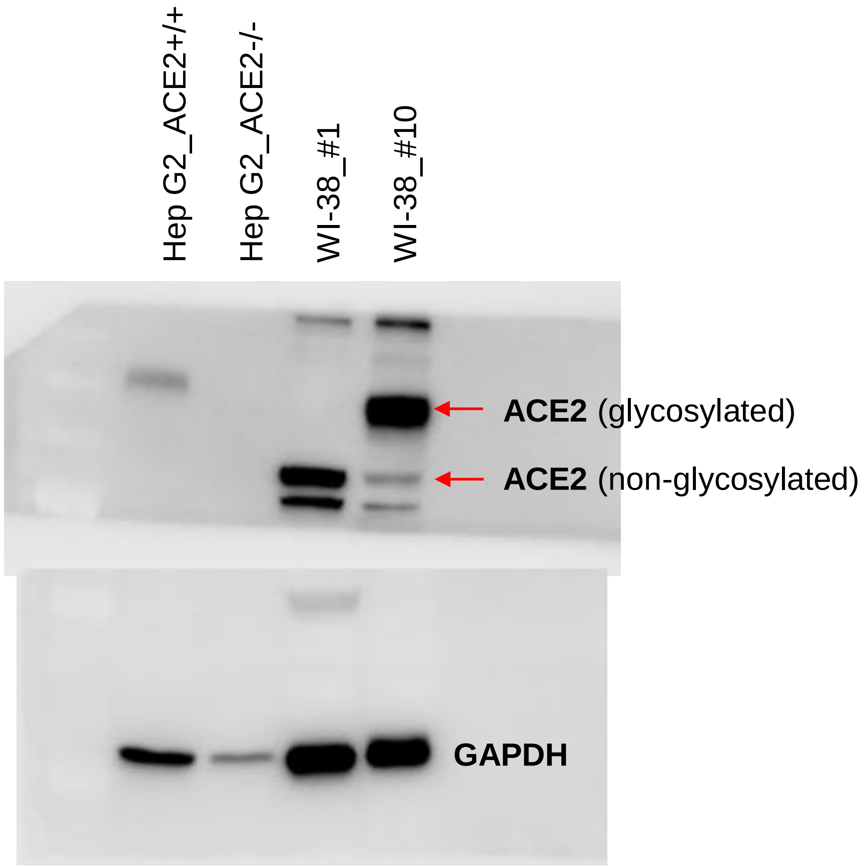

Supplement: Supplementary file 3 — Original images [file 41420_2022_876_MOESM3_ESM.pdf]
